# Unraveling the timeline of gene expression: A pseudotemporal trajectory analysis of single-cell RNA sequencing data

**DOI:** 10.12688/f1000research.134078.1

**Published:** 2023-06-15

**Authors:** Jinming Cheng, Gordon K. Smyth, Yunshun Chen

**Affiliations:** 1 Bioinformatics Division, Walter and Eliza Hall Institute of Medical Research, Melbourne, Victoria, 3052, Australia; 2 Department of Medical Biology, The University of Melbourne, Melbourne, Victoria, 3052, Australia; 3 School of Mathematics and Statistics, The University of Melbourne, Melbourne, Victoria, 3052, Australia; 4 ACRF Cancer Biology and Stem Cells Division, Walter and Eliza Hall Institute of Medical Research, Melbourne, Victoria, 3052, Australia

**Keywords:** Single-cell RNA-seq, mammary gland, trajectory analysis, time course analysis, pseudo-bulk, differential expression analysis

## Abstract

**Background:** Single-cell RNA sequencing (scRNA-seq) technologies have rapidly developed in recent years. The droplet-based single cell platforms enable the profiling of gene expression in tens of thousands of cells per sample. The goal of a typical scRNA-seq analysis is to identify different cell subpopulations and their respective marker genes. Additionally, trajectory analysis can be used to infer the developmental or differentiation trajectories of cells.

**Methods:** This article demonstrates a comprehensive workflow for performing trajectory inference and time course analysis on a multi-sample single-cell RNA-seq experiment of the mouse mammary gland. The workflow uses open-source R software packages and covers all steps of the analysis pipeline, including quality control, doublet prediction, normalization, integration, dimension reduction, cell clustering, trajectory inference, and pseudo-bulk time course analysis. Sample integration and cell clustering follows the Seurat pipeline while the trajectory inference is conducted using the monocle3 package. The pseudo-bulk time course analysis uses the quasi-likelihood framework of edgeR.

**Results:** Cells are ordered and positioned along a pseudotime trajectory that represented a biological process of cell differentiation and development. The study successfully identified genes that were significantly associated with pseudotime in the mouse mammary gland.

**Conclusions:** The demonstrated workflow provides a valuable resource for researchers conducting scRNA-seq analysis using open-source software packages. The study successfully demonstrated the usefulness of trajectory analysis for understanding the developmental or differentiation trajectories of cells. This analysis can be applied to various biological processes such as cell development or disease progression, and can help identify potential biomarkers or therapeutic targets.

## Introduction

Single-cell RNA sequencing (scRNA-seq) has emerged as a popular technique for transcriptomic profiling of samples at the single-cell level. With droplet-based methods, thousands of cells can be sequenced in parallel using next-generation sequencing platforms. ^1^^,^^2^ One of the most widely used droplet-based scRNA-seq technologies is the 10x Genomics Chromium which enables profiling transcriptomes of tens of thousands of cells per sample. ^3^ A common goal of a scRNA-seq analysis is to investigate cell types and states in heterogeneous tissues. To achieve this, various pipelines have been developed, such as *Seurat* ^4^ and the Bioconductor’s OSCA pipeline. ^5^ A typical scRNA-seq data analysis pipeline involves quality control, normalization, dimension reduction, cell clustering, and differential expression analysis.

As the cost of scRNA-seq continues to drop, more experimental studies involve replicate samples. In a multiple sample single-cell experiment, an integration method is required to investigate all cells across all samples simultaneously. This ensures that sample and batch effects are appropriately considered in visualizing and clustering cells. Popular integration methods include the Seurat’s anchor-based integration method, ^4^ Harmony, ^6^ and the MNN. ^7^

After integration and cell clustering, differential expression analysis is often performed to identify marker genes for each cell cluster. Various methods have been developed at the single-cell level for finding marker genes. ^8^^,^^9^ Recently, the pseudo-bulk method has become increasingly popular due to its superior computational efficiency and its ability to consider biological variation between replicate samples. ^10^

Trajectory inference is another popular downstream analysis that aims to study cell differentiation or cell type development. Popular software tools to perform trajectory analysis include *monocle3* ^11^ and *slingshot.* ^12^ These methods learn trajectories based on the change of gene expression and order cells along a trajectory to obtain pseudotime. ^13^^,^^14^ This allows for pseudotime-based time course analysis in single-cell experiments, which is extremely useful for investigating specific biological questions of interest.

Here we present a new single-cell workflow that integrates trajectory analysis and pseudo-bulking to execute a single-cell pseudo time course analysis. The inputs for this workflow are single-cell count matrices, such as those generated by 10x Genomic’s *cellranger.* The methods involved open source packages in R. The single-cell level analysis is performed in *Seurat*, and the trajectory analysis is conducted using *monocle3.* Once the pseudo-bulk samples are created and assigned pseudotime, a time course analysis is conducted in *edgeR.* ^15^ The analysis pipeline presented in this article can be applied to any scRNA-seq study with replicate samples.

## Description of the biological experiment

The scRNA-seq data used to demonstrate this workflow consists of five mouse mammary epithelium samples at five different stages: embryonic, early postnatal, pre-puberty, puberty and adult. The puberty sample is from the study in Pal et al. 2017, ^16^ whereas the other samples are from Pal et al. 2021. ^17^ These studies examined the stage-specific single-cell profiles in order to gain insight into the early developmental stages of mammary gland epithelial lineage. The *cellranger* count matrix outputs of these five samples are available on the GEO repository as series GSE103275 and GSE164017.

## Data preparation

### Downloading the data

The *cellranger* output of each sample consists of three key files: a count matrix in mtx.gz format, barcode information in tsv.gz format and feature (or gene) information in tsv.gz format.

The outputs of the mouse mammary epithelium at embryonic stage (E18.5), post-natal 5 days (P5), 2.5 weeks (Pre-puberty), and 10 weeks (Adult) can be downloaded from GSE164017, ^17^ whereas the output of mouse mammary epithelium at 5 weeks (Puberty) can be downloaded from GSE103275. ^16^

We first create a data directory to store all the data files.
> data_dir <- "data"
> if(**!**dir.exists(data_dir)){dir.create(data_dir, recursive=TRUE)}


We then download the barcode and count matrix files of the five samples.
> accessions <-c("GSM4994960","GSM4994962","GSM4994963","GSM2759554","GSM4994967")
> stages <- c("E18-ME", "Pre-D5-BL6", "Pre-BL6", "5wk-1", "Adult-BL6")
> file_suffixes <- c("barcodes.tsv.gz", "matrix.mtx.gz")
> for ( i in 1:length(accessions) ) {
+  for (file_suffix in file_suffixes) {
+    filename <- paste0(accessions[i],"_",stages[i],"-",file_suffix)
+    url <- paste0("http://www.ncbi.nlm.nih.gov/geo/download/?acc=",
+            accessions[i],"&","format=file&","file=",filename)
+    download.file(url=url,destfile=paste0(data_dir,"/",filename))
+  }
+ }


Since the five samples in this workflow are from two separate studies and were processed using different versions of mouse genome, the feature information is slightly different between the two runs. Here, we download the feature information of both runs. The GSM2759554_5wk-1-genes.tsv.gz file contains the feature information for the 5wk-1 sample, whereas GSE164017_features.tsv.gz contains the feature information for the other four samples.
> GSE <- c("GSE164017", "GSM2759554")
> feature_filenames <- c("GSE164017_features.tsv.gz",
+               "GSM2759554_5wk-1-genes.tsv.gz")
> for (i in 1:length(GSE) ) {
+  url <- paste0("http://www.ncbi.nlm.nih.gov/geo/download/?acc=",
+           GSE[i],"&","format=file&","file=",feature_filenames[i])
+  download.file(url=url,destfile=paste0(data_dir,"/",feature_filenames[i]))
+ }


A target information file is created to store all the sample and file information.
> samples <- c("E18.5-epi", "P5", "Pre-puberty", "Puberty", "Adult")
> targets <- data.frame(
+   samples=samples,
+   stages=stages,
+   accessions=accessions,
+   matrix.file = paste0("data/",accessions[1:5],"_",stages[1:5],"-","matrix.mtx.gz"),
+   barcode.file = paste0("data/",accessions[1:5],"_",stages[1:5],"-","barcodes.tsv.gz"),
+   feature.file = paste0("data/",feature_filenames[c(1,1,1,2,1)]))
> targets

      samples     stages accessions                              matrix.file
1   E18.5-epi     E18-ME GSM4994960     data/GSM4994960_E18-ME-matrix.mtx.gz
2          P5 Pre-D5-BL6 GSM4994962 data/GSM4994962_Pre-D5-BL6-matrix.mtx.gz
3 Pre-puberty    Pre-BL6 GSM4994963    data/GSM4994963_Pre-BL6-matrix.mtx.gz
4     Puberty      5wk-1 GSM2759554      data/GSM2759554_5wk-1-matrix.mtx.gz
5       Adult  Adult-BL6 GSM4994967  data/GSM4994967_Adult-BL6-matrix.mtx.gz
                                barcode.file                       feature.file
1     data/GSM4994960_E18-ME-barcodes.tsv.gz     data/GSE164017_features.tsv.gz
2 data/GSM4994962_Pre-D5-BL6-barcodes.tsv.gz     data/GSE164017_features.tsv.gz
3    data/GSM4994963_Pre-BL6-barcodes.tsv.gz     data/GSE164017_features.tsv.gz
4      data/GSM2759554_5wk-1-barcodes.tsv.gz data/GSM2759554_5wk-1-genes.tsv.gz
5  data/GSM4994967_Adult-BL6-barcodes.tsv.gz     data/GSE164017_features.tsv.gz


### Reading in the data

The downloaded *cellranger* outputs of all the samples can be read in one-by-one using the read10X function in the *edgeR* package. First, a DGElist object is created for each sample, which is then consolidated into a single DGElist object by merging them altogether.
> library(edgeR)
> dge_all <- list()
> for ( i in 1:5 ) {
+   y <- read10X(mtx = targets**$**matrix.file[i],
+     barcodes = targets**$**barcode.file[i], genes = targets**$**feature.file[i])
+   y**$**samples**$**group <- targets**$**samples[i]
+   colnames(y) <- paste0(targets**$**accessions[i],"-",y**$**samples**$**Barcode)
+   y**$**genes**$**Ensembl_geneid <- rownames(y)
+   y**$**genes <- y**$**genes[,c("Ensembl_geneid","Symbol")]
+   y <- y[**!**duplicated(y**$**genes**$**Symbol),]
+   rownames(y) <- y**$**genes**$**Symbol
+   dge_all[[i]] <- y
+ }
> rm(y)
> common.genes <- Reduce(intersect, lapply(dge_all, rownames))
> for(i in 1:5) dge_all[[i]] <- dge_all[[i]][common.genes, ]
> dge_merged <- do.call("cbind", dge_all)


The levels of group in the sample information data frame are reordered and renamed from the early embryonic stage to the late adult stage.
> dge_merged**$**samples**$**group <- factor(dge_merged**$**samples**$**group, levels=samples)


The number of genes, the total number of cells, and the number of cells in each sample are shown below.
> dim(dge_merged)

[1] 26589 33735

> table(dge_merged**$**samples**$**group)

  E18.5-epi         P5 Pre-puberty      Puberty      Adult
       6969       3886        4183         5428      13269


## Single-cell RNA-seq analysis

### Quality control

Quality control (QC) is essential for single-cell RNA-seq data analysis. Common choices of QC metrics include number of expressed genes or features, library size, and proportion of reads mapped to mitochondrial genes in each cell. The number of expressed genes and mitochondria read percentage in each cell can be calculated as follows.
> dge_merged**$**samples**$**num_exp_gene <- colSums(dge_merged**$**counts>0)
> mito_genes <- rownames(dge_merged)[grep("^mt-",rownames(dge_merged))]
> dge_merged**$**samples**$**mito_percentage <-
+  colSums(dge_merged**$**counts[mito_genes,])/
+  colSums(dge_merged**$**counts)*100


**Figure 1.**
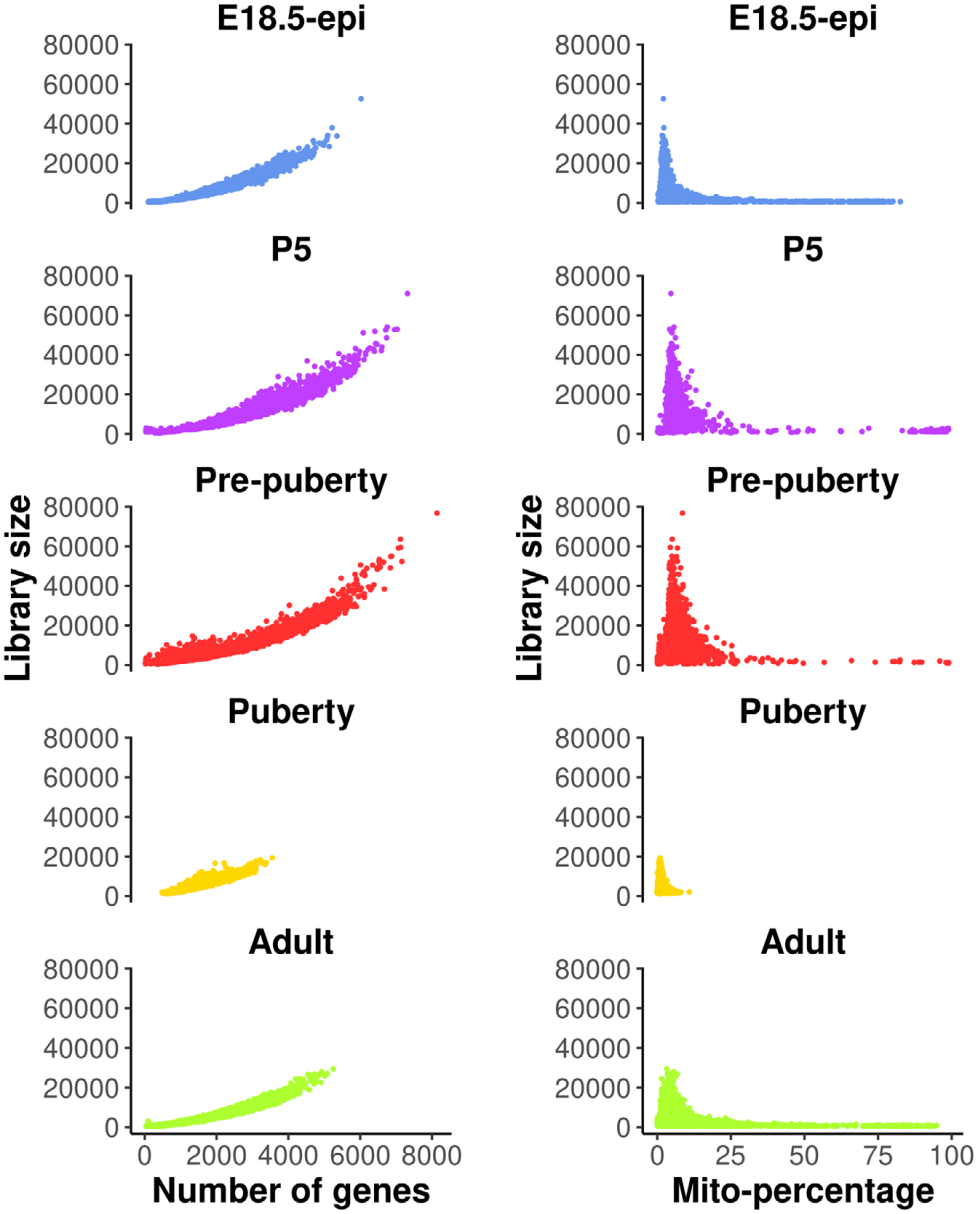
Scatter plots of quality control metrics across all the samples. The plots on the left show library size vs number of genes detected, whereas those on the right show library size vs mitochondria read percentage.

These QC metrics can be visualized in the following scatter plots ( Figure 1).
> library(ggplot2)
> my_theme_ggplot <- theme_classic() +
+  theme(axis.text=element_text(size=12),
+      axis.title=element_text(size=15,face="bold"),
+      plot.title=element_text(size=15,face="bold",hjust=0.5),
+      plot.margin=margin(0.5, 0.5, 0.5, 0.5, "cm"))
> my_theme_facet <-
+  theme(strip.background=element_rect(colour="white",fill="white"),
+      strip.text=element_text(size=15, face="bold",color="black"))
> my_colors_15 <- c("cornflowerblue", "darkorchid1", "firebrick1", "gold",
+            "greenyellow", "mediumspringgreen", "mediumturquoise",
+            "orange1", "pink", "deeppink3", "violet", "magenta",
+            "goldenrod4", "cyan", "gray90")
> p1 <- ggplot(data = dge_merged**$**samples,
+         aes(x=num_exp_gene, y=lib.size, color = group ) ) +
+     geom_point(size=0.5, show.legend=FALSE) +
+     facet_wrap(group~., ncol=1) +
+     scale_color_manual(values=my_colors_15 ) +
+     labs(x="Number of genes", y="Library size") +
+     my_theme_ggplot + my_theme_facet
> p2 <- ggplot(data = dge_merged**$**samples,
+         aes(x = mito_percentage, y=lib.size, color = group ) ) +
+     geom_point(size = 0.5, show.legend = FALSE) +
+     facet_wrap(group~., ncol=1) +
+     scale_color_manual(values=my_colors_15) +
+     labs(x="Mito-percentage", y="Library size") +
+     my_theme_ggplot + my_theme_facet
> patchwork::wrap_plots(p1, p2, ncol=2)


Cells with a very low number of genes (<500), as well as high mitochondria read percentage (>10%), are considered of low quality and hence are removed from the analysis. Cells expressing a large number of genes are also removed as they are likely to be doublets. Different thresholds are selected for different samples based on the distribution of the number of genes expressed. Here, we choose 5000, 6000, 6000, 3000, 4000 for E18.5-epi, P5, pre-puberty, puberty and adult samples, respectively. In this workflow, most of the single-cell analysis is conducted using the**** *Seurat* package. A list of five Seurat objects are first created to store the data after QC.
> library(Seurat)
> n_genes_max <- c(5000, 6000, 6000, 3000, 4000)
> data_seurat <- list()
> for (i in 1:5) {
+  sel <- dge_merged**$**samples**$**group == samples[i]
+  y <- dge_merged[, sel]
+  data_seurat[[i]] <- CreateSeuratObject( counts=y**$**counts,
+     meta.data=y**$**samples, min.cells=3, min.features=200,
+     project=samples[i] )
+  data_seurat[[i]] <- subset( data_seurat[[i]],
+     subset = (nFeature_RNA > 500) & (nFeature_RNA < n_genes_max[i]) &
+           (mito_percentage < 10) )
+ }
> names(data_seurat) <- samples


### Standard Seurat analysis of individual sample

A standard Seurat analysis is performed for each individual sample. In particular, the data of each sample is first normalized by the default log normalization method in NormalizeData. The top 2000 highly variable genes are identified by FindVariableFeatures. The normalized data of the 2000 highly variable genes are scaled by ScaleData to have a mean of 0 and a variance of 1. The principal component analysis (PCA) dimension reduction is performed on the highly variable genes by RunPCA. Uniform manifold approximation and projection (UMAP) dimension reduction is performed on the first 30 PCs by RunUMAP. Cell clustering is performed by FindNeighbors and FindClusters. The cell clustering resolution is set at 0.1, 0.1, 0.2, 0.2 and 0.2 for E18.5-epi, P5, pre-puberty, puberty and adult, respectively.
> data_seurat <- lapply(data_seurat, NormalizeData)
> data_seurat <- lapply(data_seurat, FindVariableFeatures, nfeatures=2000)
> data_seurat <- lapply(data_seurat, ScaleData)
> data_seurat <- lapply(data_seurat, RunPCA, verbose = FALSE)
> data_seurat <- lapply(data_seurat, RunUMAP, reduction = "pca", dims = 1:30)
> data_seurat <- lapply(data_seurat, FindNeighbors, reduction="pca", dims=1:30)
> resolutions <- c(0.1, 0.1, 0.2, 0.2, 0.2)
> for(i in 1:5)
+   data_seurat[[i]] <- FindClusters(data_seurat[[i]],
+      resolution=resolutions[i], verbose=FALSE)


### Removing potential doublets and non-epithelial cells

Although high-throughput droplet-based single-cell technologies can accurately capture individual cells, there are instances where a single droplet may contain two or more cells, which are known as doublets or multiplets. Here we use the *scDblFinder* package ^18^ to further remove potential doublets. To do that, each Seurat object in the list is first converted into a SingleCellExperiment object using the as.SingleCellExperiment function in *Seurat.* Then the scDblFinder function in the *scDblFinder* package is called to predict potential doublets on each SingleCellExperiment object. The scDblFinder output for each sample is stored in the corresponding Seurat object.
> library(scDblFinder)
> for (i in 1:5) {
+  sce <- as.SingleCellExperiment(DietSeurat(data_seurat[[i]],
+     graphs=c("pca","umap")) )
+  set.seed(42)
+  sce <- scDblFinder(sce)
+  data_seurat[[i]]**$**db_score <- sce**$**scDblFinder.score
+  data_seurat[[i]]**$**db_type <- factor( sce**$**scDblFinder.class,
+     levels=c("singlet", "doublet") )
+ }


**Figure 2.**
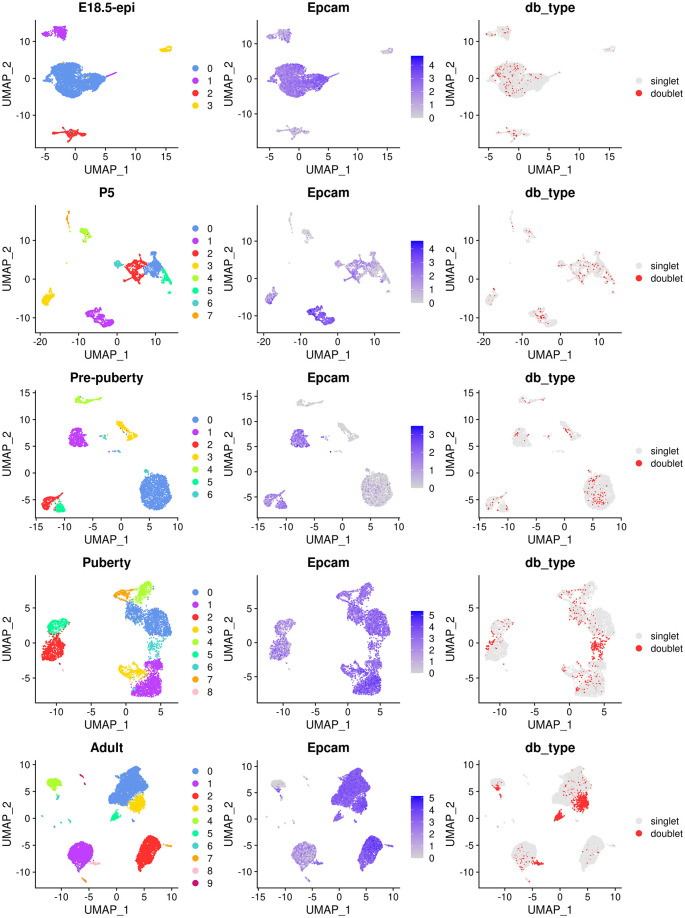
UMAP visualization of each individual samples. The UMAP plots, in sequence from the top row to the bottom row, correspond to E18.5-epi, P5, Pre-puberty, Puberty, and Adult, respectively. In each row, cells are coloured by cluster on the left, by Epcam expression level in the middle, and by doublet prediction on the right.

The main object of this single-cell experiment is to examine the early developmental stages of the mouse epithelial mammary gland. Therefore, we focus on epithelial cells for the rest of the analysis. We use the *Epcam* gene to identify epithelial cell clusters in each sample. The cell clustering, the expression level of *Epcam* and doublet prediction results of each sample are shown below ( Figure 2).
> p1 <- lapply(data_seurat,function(x){DimPlot(x, pt.size=0.1, cols=my_colors_15) +
+          ggtitle(x**$**group[1]) + theme(plot.title=element_text(hjust=0.5))})
> p2 <- lapply(data_seurat, FeaturePlot, feature="Epcam", pt.size=0.1)
> p3 <- lapply(data_seurat, DimPlot, group.by="db_type", pt.size=0.1,
+         cols=c("gray90", "firebrick1"))
> patchwork::wrap_plots(c(p1,p2,p3), nrow=5, byrow=FALSE)


By examining the expression level of the *Epcam* gene, we select the following clusters in each sample as the epithelial cell population.
> epi_clusters <- list(
+  "E18.5-epi" = 0,
+  "P5" = c(1,3),
+  "Pre-puberty" = c(0:2, 5),
+  "Puberty" = 0:6,
+  "Adult" = 0:3
+ )


Cells that are non-epithelial and those identified as potential doublets by *scDblFinder* are excluded from the subsequent analysis. The cellular barcodes of the remaining epithelial cells from each sample are stored in the list object called epi_cells. The respective number of epithelial cells that are retained for each sample is shown below.
> epi_cells <- list()
> for (i in samples) {
+  epi_cells[[i]] <- rownames(
+   subset(data_seurat[[i]]@meta.data,
+    (db_type == "singlet") & (seurat_clusters %in% epi_clusters[[i]])))
+ }
> do.call(c, lapply(epi_cells, length))

  E18.5-epi          P5 Pre-puberty      Puberty      Adult
       4343        1140        2546         4706       9341


## Data integration

### Integrating epithelial cells of five samples

Since we have five individual scRNA-seq samples, conducting an integration analysis is necessary to explore all cells across these samples simultaneously. In this workflow, we use the anchor-based method in the *Seurat* package for integration. A Seurat object is first created from the merged DGEList object of epithelial cells using CreateSeuratObject function without filtering any cells ( min.features is set to 0).
> epi_cells <- do.call(c, epi_cells)
> dge_merged_epi <- dge_merged[, epi_cells]
> seurat_merged <- CreateSeuratObject(counts = dge_merged_epi**$**counts,
+              meta.data = dge_merged_epi**$**samples,
+              min.cells = 3, min.features = 0, project = "mammary_epi")


Then the Seurat object is split into a list of five Seurat objects, where each object corresponds to one of the five samples. For each sample, the log normalization method is applied to normalize the raw count by NormalizeData, and highly variable genes are identified by FindVariableFeatures.
> seurat_epi <- SplitObject(seurat_merged, split.by = "group")
> seurat_epi <- lapply(seurat_epi, NormalizeData)
> seurat_epi <- lapply(seurat_epi, FindVariableFeatures, nfeatures = 2000)


The feature genes used for integration are chosen by SelectIntegrationFeatures, and these genes are used to identify anchors for integration by FindIntegrationAnchors. The integration process is performed by IntegrateData based on the identified anchors.
> anchor_features <- SelectIntegrationFeatures(seurat_epi,
+              nfeatures = 2000, verbose = FALSE)
> anchors <- FindIntegrationAnchors(seurat_epi, verbose = FALSE,
+              anchor.features = anchor_features)
> seurat_int <- IntegrateData(anchors, verbose = FALSE)


The integrated data are then scaled to have a mean of 0 and a variance of 1 by ScaleData. PCA is performed on the scaled data using RunPCA, followed by UMAP using RunUMAP. Cell clusters of the integrated data are identified by using FindNeighbors and FindClusters.
> DefaultAssay(seurat_int) <- "integrated"
> seurat_int <- ScaleData(seurat_int, verbose = FALSE)
> seurat_int <- RunPCA(seurat_int, npcs = 30, verbose = FALSE)
> seurat_int <- RunUMAP(seurat_int, reduction = "pca",
+           dims = 1:30, verbose = FALSE)
> seurat_int <- FindNeighbors(seurat_int, dims = 1:30, verbose = FALSE)
> seurat_int <- FindClusters(seurat_int, resolution = 0.2, verbose = FALSE)


**Figure 3.**
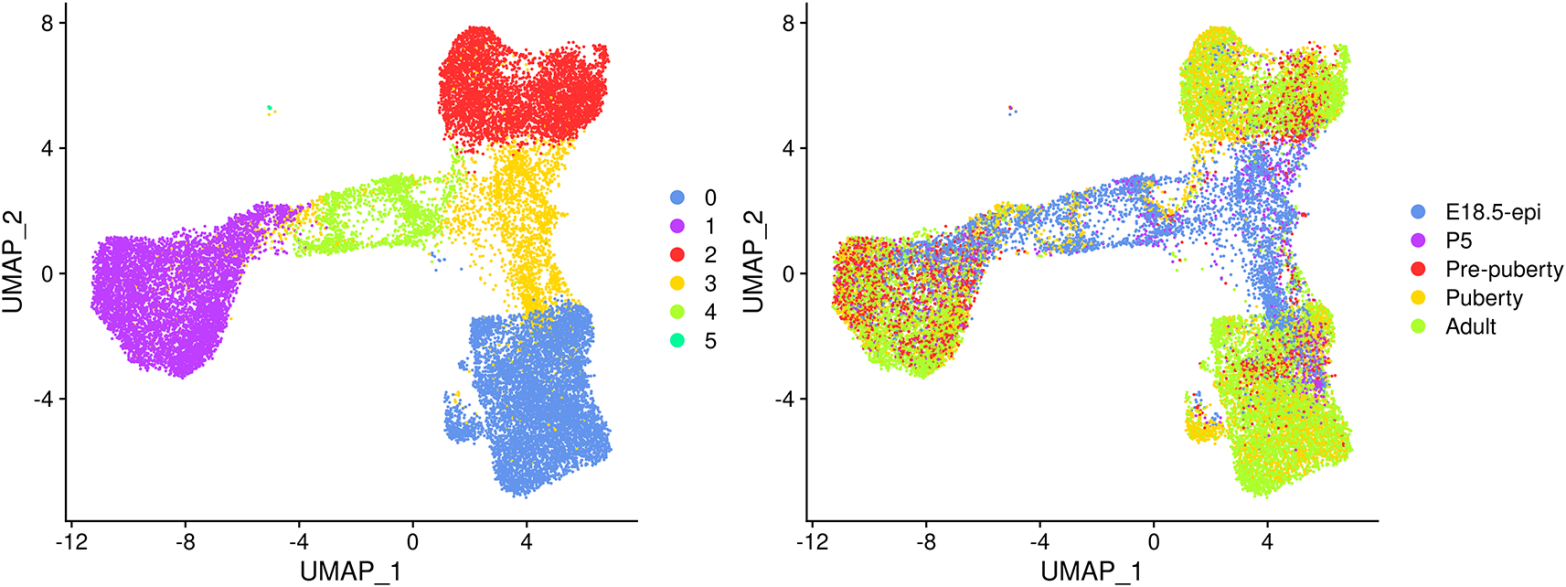
UMAP visualization of the integrated data. Cells are coloured by cluster on the left and by original sample on the right.

UMAP plots are generated to visualize the integration and cell clustering results ( Figure 3). The UMAP plot indicates the presence of three major cell clusters (cluster 0, 1, and 2), which are bridged by intermediate clusters located in between them. Cells at the later stages largely dominate the three major cell clusters, while cells at the earlier stages are predominantly present in the intermediate clusters in the middle.
> seurat_int**$**group <- factor(seurat_int**$**group, levels = samples)
> p1 <- DimPlot(seurat_int, pt.size = 0.1, cols = my_colors_15)
> p2 <- DimPlot(seurat_int, pt.size = 0.1, group.by = "group",
+       shuffle = TRUE, cols = my_colors_15) + labs(title="")
> p1 | p2


### Cell type identification

**Figure 4.**
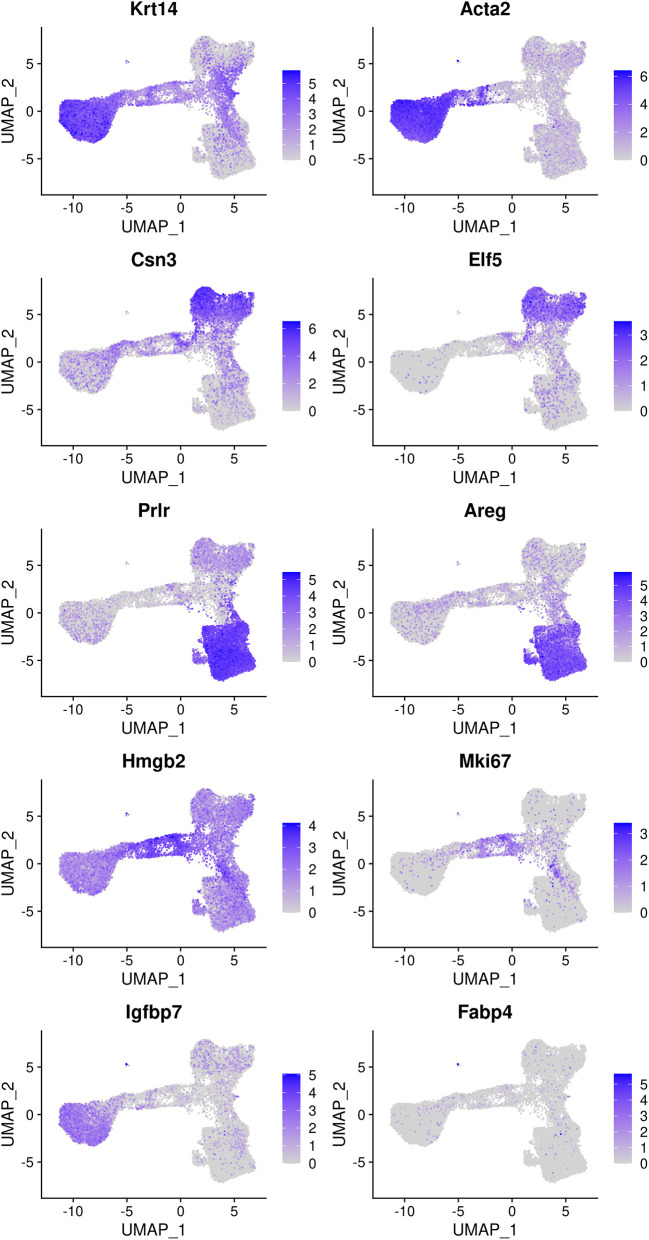
Feature plots of the integrated data. Genes from the top row to the bottom rows are the markers of basal, LP, ML, cycling, and stromal cells, respectively.

The mammary gland epithelium consists of three major cell types: basal myoepithelial cells, luminal progenitor (LP) cells and mature luminal (ML) cells. These three major epithelial cell populations have been well studied in the literature. By examining the classic marker genes of the three cell types, we are able to identify basal, LP and ML cell populations in the integrated data ( Figure 4). Here we use *Krt14* and *Acta2* for basal, *Csn3* and *Elf5* for LP, and *Prlr* and *Areg* for ML. We also examine the expression level of *Hmgb2* and *Mki67* as they are typical markers for cycling cells and the expression level of *Igfbp7* and *Fabp4* as they are marker genes for stromal cells.
> markers <- c("Krt14", "Acta2", "Csn3","Elf5", "Prlr","Areg",
+              "Hmgb2", "Mki67", "Igfbp7","Fabp4")
> DefaultAssay(seurat_int) <- "RNA"
> FeaturePlot(seurat_int, order = TRUE, pt.size = 0.1, features = markers, ncol = 2)


Based on the feature plots, cluster 1, cluster 2 and cluster 0 represent the basal, LP and ML cell populations, respectively. Cluster 4 mainly consists of cycling cells, whereas cluster 3 seems to be a luminal intermediate cell cluster expressing both LP and ML markers. Cluster 5 consists of a few non-epithelial (stromal) cells that have not been filtered out previously.

The number of cells in each cluster for each sample is shown below.
> tab_number <- table(seurat_int**$**group, seurat_int**$**seurat_clusters)
> tab_number

                0    1    2    3    4   5
 E18.5-epi   171  878   29 2341  921   3
 P5          272  347   47  351  120   3
 Pre-puberty 381 1590  482   64   23   6
 Puberty    1894  986 1535   20  265   6
 Adult      4281 2495 2362  171   32   0


The proportion of cells in each cluster is calculated for each sample to compare the variation in cell composition across different stages.
> tab_ratio <- round(100*tab_number/rowSums(tab_number), 2)
> tab_ratio <- as.data.frame.matrix(tab_ratio)
> tab_ratio

                0    1     2     3     4    5
E18.5-epi    3.94 20.2  0.67 53.90 21.21 0.07
P5          23.86 30.4  4.12 30.79 10.53 0.26
Pre-puberty 14.96 62.5 18.93  2.51  0.90 0.24
Puberty     40.25 20.9 32.62  0.42  5.63 0.13
Adult       45.83 26.7 25.29  1.83  0.34 0.00


**Figure 5.**
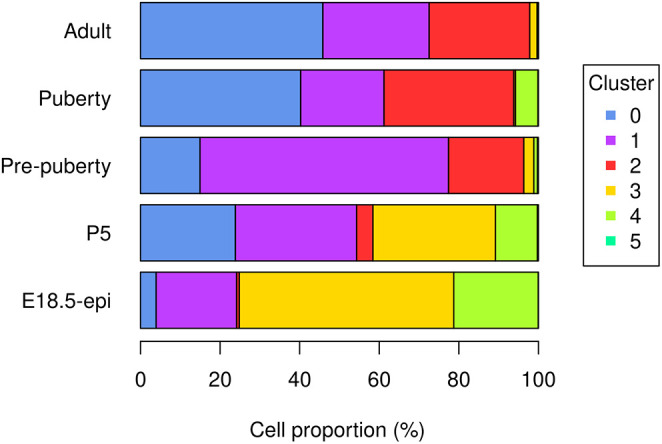
Bar plot of cell proportion of each cluster in each sample.

The bar plot ( Figure 5) shows the proportion of different cell types in samples at different developmental stages. Specifically, the proportion of basal cells (purple) demonstrates an ascending trend from E18.5 to pre-puberty stage, after which it declines towards adult stage. The LP cell proportion (red) rises from E18.5 to puberty stage, followed by a slight dip at adult stage. Although the proportion of ML cells (blue) is higher at P5 than pre-puberty stage, it shows an overall increasing trend. Cycling cells (green) constitute the highest proportion at E18.5 stage, but decrease to a smaller proportion at pre-puberty stage, with a slight increase at puberty stage, and subsequently, they reduce to a negligible proportion at adult stage. The augmented cycling cell proportion at puberty stage aligns with the ductal morphogenesis characteristics of the mammary gland. The luminal intermediate cell proportion (yellow) displays a decreasing trend from E18.5 stage to adult stage.
> par(mar=c(5, 7, 1, 7), xpd=TRUE)
> barplot(t(tab_ratio), col=my_colors_15, xlab="Cell proportion (%)",
+   horiz = TRUE, las=1)
> legend("right", inset = c(-0.3,0), legend = 0:5, pch = 15,
+   col=my_colors_15, title="Cluster")


## Trajectory analysis with monocle3

### Constructing trajectories and pseudotime

Many biological processes manifest as a dynamic sequence of alterations in the cellular state, which can be estimated through a “trajectory” analysis. Such analysis is instrumental in detecting the shifts between different cell identities and modeling gene expression dynamics. By treating single-cell data as a snapshot of an uninterrupted process, the analysis establishes the sequence of cellular states that forms the process trajectory. The arrangement of cells along these trajectories can be interpreted as pseudotime.

Here, we use the *monocle3* package to infer the development trajectory in the mouse mammary gland epithelial cell population. The Seurat object of the integrated data is first converted into a cell_data_set object to be used in *monocle3.*
> library(monocle3)
> cds_obj <- SeuratWrappers::as.cell_data_set(seurat_int)


*monocle3* re-clusters cells to assign them to specific clusters and partitions, which are subsequently leveraged to construct trajectories. If multiple partitions are used, each partition will represent a distinct trajectory. The calculation of pseudotime, which indicates the distance between a cell and the starting cell in a trajectory, is conducted during the trajectory learning process. These are done using the cluster_cells and learn_graph functions. To obtain a single trajectory and avoid a loop structure, both use_partition and close_loop are turned off in learn_graph.
> set.seed(42)
> cds_obj <- cluster_cells(cds_obj)
> cds_obj <- learn_graph(cds_obj, use_partition=FALSE, close_loop=FALSE)


### Visualizing trajectories and pseudotime

**Figure 6.**
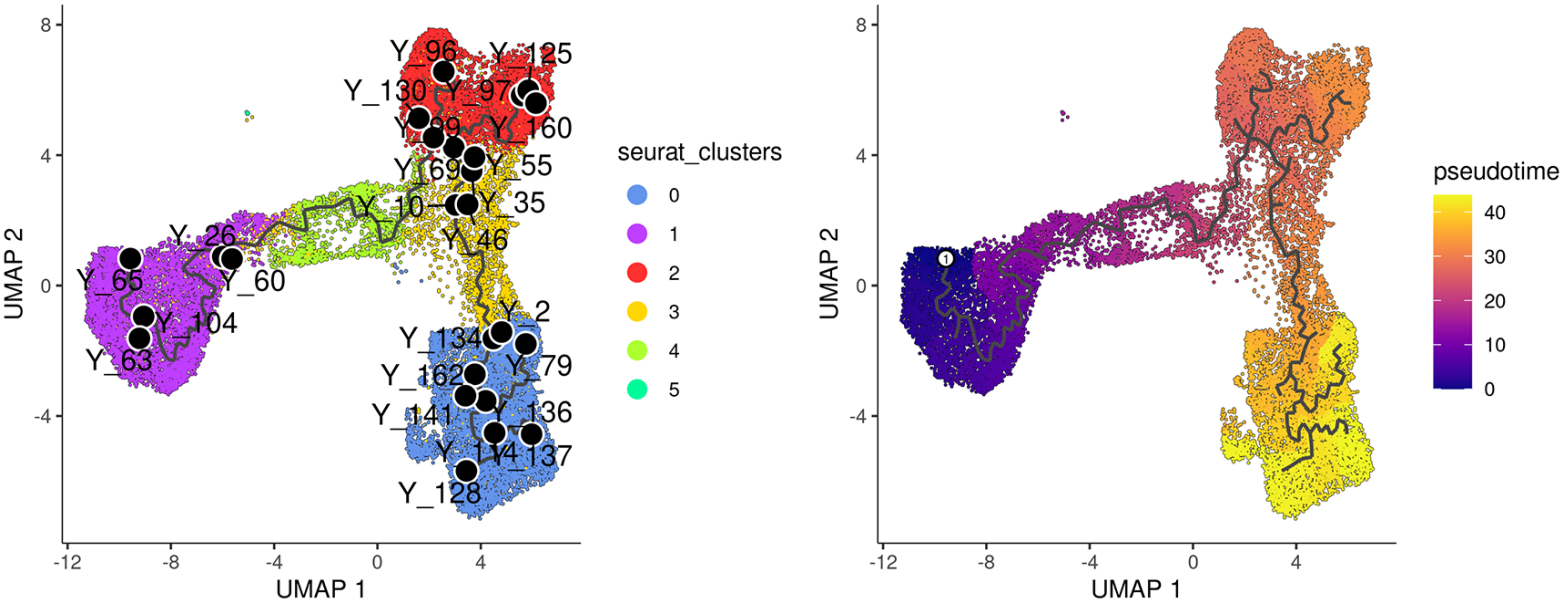
UMAP visualization of trajectory and pseudotime computed by monocle3. Cells are coloured by cluster on the left and by pseudotime on the right.

The plot_cells function of *monocle3* is used to generate a trajectory plot that superimposes the trajectory information onto the UMAP representation of the integrated data. By adjusting the label_principal_points parameter, the names of roots, leaves, and branch points can be displayed. Cells in the trajectory UMAP plot ( Figure 6) on the left are colored by cell cluster identified in the previous Seurat integration analysis.

Along the *monocle3* trajectory analysis, several nodes are identified and marked with black circular dots on the resulting plot, representing key points along the trajectories. To establish the order of cells and calculate their corresponding pseudotime, it is necessary to select a starting node from among the identified nodes. For this analysis, node “Y_65” in the basal population (cluster 1) was selected as the starting node, as mammary stem cells are known to be enriched in the basal population and give rise to LP and ML cells in the epithelial lineage. ^19^ It should be noted that node numbers may vary depending on the version of monocle3 used.

The cells are then ordered and assigned pseudotime values by the order_cells function in *monocle3.* The resulting pseudotime information can be visualized on the UMAP plot by using the plot_cells function, as demonstrated in the UMAP plot on the right ( Figure 6).
> p1 <- plot_cells(cds_obj, color_cells_by="seurat_clusters",
+           group_label_size=4, graph_label_size=3,
+           label_cell_groups=FALSE, label_principal_points=TRUE,
+           label_groups_by_cluster=FALSE) +
+    scale_color_manual(values = my_colors_15)
> cds_obj <- order_cells(cds_obj, root_pr_nodes="Y_65")
> p2 <- plot_cells(cds_obj, color_cells_by="pseudotime",
+           label_groups_by_cluster=FALSE, label_leaves=FALSE,
+           label_branch_points=FALSE)
> p1 | p2


The pseudotime function in *monocle3* allows users to extract the pseudotime values of the cells from a cell_data_set object. This information can then be stored in the metadata of the Seurat object for further analysis.
> seurat_int**$**pseudotime <- pseudotime(cds_obj)


## Pseudo-bulk time course analysis with edgeR

### Constructing pseudo-bulk profiles

After obtaining the pseudotime of each cell, we proceed to a time course analysis to identify genes that change significantly along the pseudotime. Our approach involves creating pseudo-bulk samples using a pseudo-bulking approach and performing an *edgeR*-style time course analysis.

To create the pseudo-bulk samples, read counts are aggregated for all cells with the same combination of sample and cluster. The number of cells used to construct each pseudo-bulk sample is added to the sample metadata. The average pseudotime of all cells in each pseudo-bulk sample is used as the pseudotime for that sample.
> y <- dge_merged[, colnames(seurat_int)]
> y**$**samples <- cbind(y**$**samples[, 1:3],
+   seurat_int@meta.data[, c("seurat_clusters", "pseudotime")])
> sample_cluster <- paste0(y**$**samples**$**group, "_C", y**$**samples**$**seurat_clusters)
> avg_pseudotime <- tapply(y**$**samples**$**pseudotime, sample_cluster, mean)
> cell_number <- table(sample_cluster)
> y <- sumTechReps(y, ID = sample_cluster)
> y**$**samples**$**pseudotime <- avg_pseudotime[colnames(y)]
> y**$**samples**$**cell_number <- cell_number[colnames(y)]


The Entrez gene IDs are added to the gene information. Genes with no valid Entrez gene IDs are removed from the downstream analysis.
> library(org.Mm.eg.db)
> entrez_id <- select(org.Mm.eg.db, keys = y**$**genes**$**Symbol,
+         columns = c("ENTREZID", "SYMBOL"), keytype = "SYMBOL")
> y**$**genes**$**ENTREZID <- entrez_id**$**ENTREZID
> y <- y[**!**is.na(y**$**genes**$**ENTREZID), ]


The samples are ordered by average pseudotime for the following analysis.
> y <- y[, order(y**$**samples**$**pseudotime)]


### Filtering and normalization

We now proceed to the standard *edgeR* analysis pipeline, which starts with filtering and normalization. The sample information, such as library sizes, average pseudotime and cell numbers, are shown below.
> y**$**samples[, c("lib.size", "pseudotime", "cell_number")]

               lib.size pseudotime cell_number
Pre-puberty_C1 11886898       4.65        1590
Adult_C1        9285265       4.77        2495
P5_C1           2680089       6.41         347
Puberty_C1      3112796       6.48         986
E18.5-epi_C1    8084434      10.16         878
E18.5-epi_C5       5179      15.61           3
P5_C5             24491      15.61           3
Pre-puberty_C5    57834      15.61           6
Puberty_C5        12278      15.61           6
Adult_C4         204212      19.25          32
E18.5-epi_C4   11725731      19.31         921
P5_C4           1917228      19.68         120
Puberty_C4      1860770      19.72         265
Pre-puberty_C4   289370      22.10          23
E18.5-epi_C3   15806768      28.03        2341
Puberty_C2      5841364      28.62        1535
P5_C3           3862152      28.94         351
E18.5-epi_C2     167242      29.14          29
Adult_C2        8320630      29.66        2362
P5_C2            347365      29.67          47
Pre-puberty_C2  4625160      30.51         482
Puberty_C3        63722      31.73          20
Pre-puberty_C3  1432336      32.64          64
Adult_C3         997670      33.99         171
Pre-puberty_C0  6024872      38.90         381
E18.5-epi_C0     990670      39.64         171
Adult_C0       27621462      40.44        4281
P5_C0           3113053      40.68         272
Puberty_C0      8806924      41.09        1894


To ensure the reliability of the analysis, it is recommended to remove pseudo-bulk samples that are constructed from a small number of cells. We suggest each pseudo-bulk sample should contain at least 30 cells. In this analysis, we identified seven pseudo-bulk samples that were constructed with less than 30 cells and removed them form the analysis.
> keep_samples <- y**$**samples**$**cell_number > 30
> y <- y[, keep_samples]


Genes with very low count number are also removed from the analysis. This is performed by the filterByExpr function in *edgeR.*
> keep_genes <- filterByExpr(y)
> y <- y[keep_genes, , keep.lib.sizes=FALSE]


The number of genes and samples after filtering are shown below.
> dim(y)
[1] 11550   22


Normalization is performed by the trimmed mean of M values (TMM) method ^20^ implemented in the calcNormFactors function in *edgeR.*
> y <- calcNormFactors(y)


**Figure 7.**
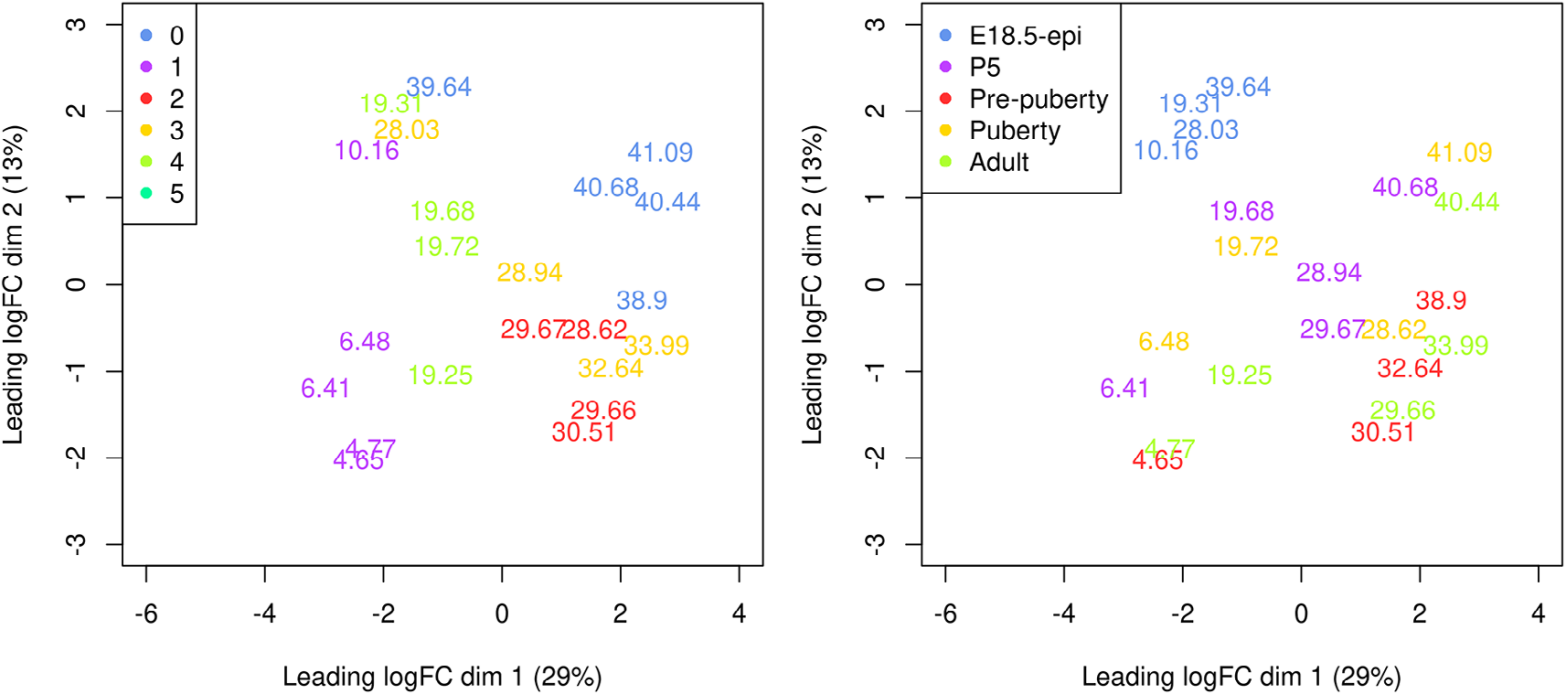
Multi-dimensional scaling (MDS) plot of the pseudo-bulk samples labelled by pseudotime. Samples are coloured by original cell cluster on the left and by developmental stage on the right.

A Multi-dimensional scaling (MDS) plot serves as a valuable diagnostic tool for investigating the relationship among samples. MDS plots are produced using the plotMDS function in *edgeR* ( Figure 7).
> par(mar = c(5.1, 5.1, 2.1, 2.1), mfrow=c(1,2))
> cluster <- y**$**samples**$**seurat_clusters
> group <- y**$**samples**$**group
> plotMDS(y, labels = round(y**$**samples**$**pseudotime, 2),
+   xlim=c(-6,4), ylim=c(-3,3), col=my_colors_15[cluster])
> legend("topleft", legend=levels(cluster), col=my_colors_15, pch=16)
> plotMDS(y, labels = round(y**$**samples**$**pseudotime, 2),
+   xlim=c(-6,4), ylim=c(-3,3), col=my_colors_15[group])
> legend("topleft", legend=levels(group), col=my_colors_15, pch=16)


On the MDS plot, pseudo-bulk samples derived from the same cell cluster are close to each other. The samples are positioned in ascending order of pseudotime from left to right, suggesting a continuous shift in the gene expression profile throughout the pseudotime.

### Design matrix

The aim of a time course experiment is to examine the relationship between gene abundances and time points. Assuming gene expression changes smoothly over time, we use a natural cubic spline with degrees of freedom of 3 to model gene expression along the pseudotime. The spline design matrix is generated by ns function in *splines.* The design matrix is also reformed so that the first column represents the linear trend.
> t1 <- y**$**samples**$**pseudotime
> X <- splines::ns(as.numeric(t1),df = 3)
> A <- cbind(1,t1,X)
> QR <- qr(A)
> r <- QR**$**rank
> R_rank <- QR**$**qr[1:r,1:r]
> Z <- t(backsolve(R_rank,t(A),transpose=TRUE))
> Z <- Z[,-1]
> design <- model.matrix(~ Z)
> design

   (Intercept)      Z1      Z2      Z3
1            1 -0.3593 -0.0550 -0.3206
2            1 -0.3572 -0.0572 -0.3116
3            1 -0.3285 -0.0887 -0.1837
4            1 -0.3271 -0.0901 -0.1780
5            1 -0.2626 -0.1489  0.0887
6            1 -0.1034 -0.0918  0.4047
7            1 -0.1024 -0.0900  0.4042
8            1 -0.0958 -0.0775  0.4002
9            1 -0.0951 -0.0761  0.3997
10           1  0.0505  0.2625  0.0541
11           1  0.0609  0.2746  0.0262
12           1  0.0666  0.2796  0.0118
13           1  0.0790  0.2862 -0.0180
14           1  0.0792  0.2863 -0.0185
15           1  0.0940  0.2854 -0.0491
16           1  0.1313  0.2389 -0.1021
17           1  0.1551  0.1804 -0.1195
18           1  0.2410 -0.1584 -0.1106
19           1  0.2540 -0.2199 -0.1034
20           1  0.2682 -0.2885 -0.0947
21           1  0.2722 -0.3084 -0.0922
22           1  0.2794 -0.3435 -0.0876
attr(,"assign")
[1] 0 1 1 1


### Dispersion estimation

**Figure 8.**
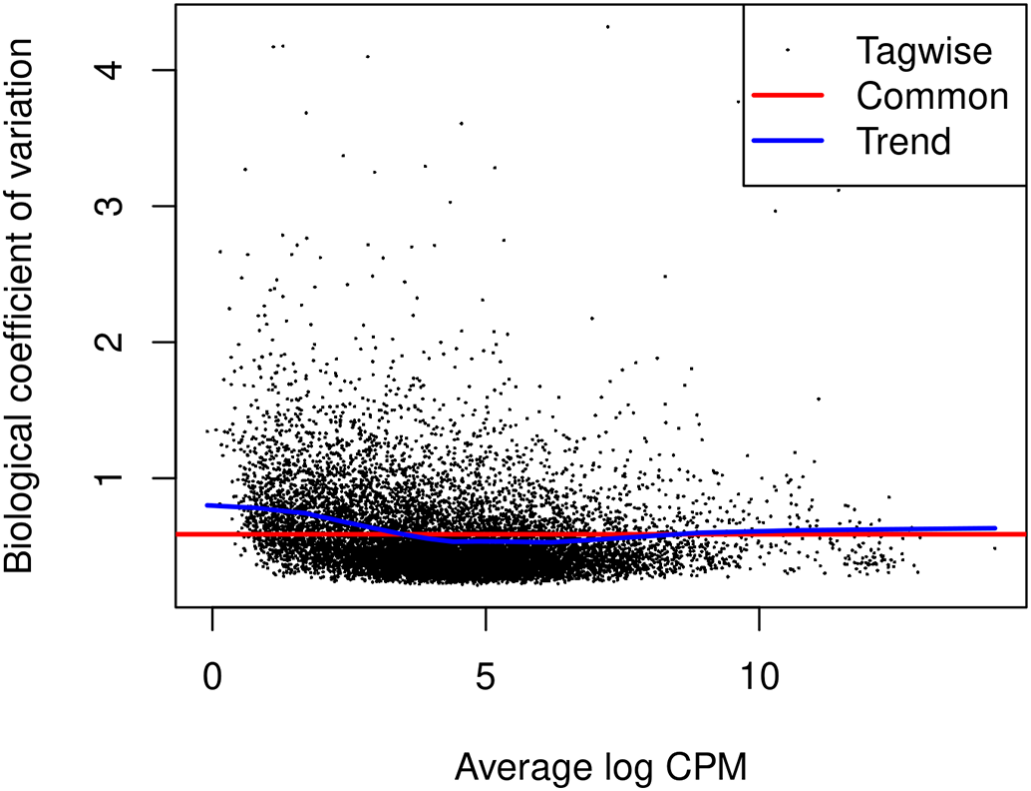
A scatter plot of the biological coefficient of variation (BCV) against the average abundance of each gene. The square-root estimates of the common, trended and gene-wise NB dispersions are shown.

The *edgeR* package uses negative binomial (NB) distribution to model read counts of each gene across all the sample. The NB dispersions are estimated by the estimateDisp function. The estimated common, trended and gene-specific dispersions can be visualized by plotBCV ( Figure 8).
> y <- estimateDisp(y, design)
> sqrt(y**$**common.dispersion)

[1] 0.588

> plotBCV(y)


The NB model can be extended with quasi-likelihood (QL) methods to account for gene-specific variability from both biological and technical sources. ^21^^,^^22^ Note that only the trended NB dispersion is used in the QL method. The gene-specific variability is captured by the QL dispersion.

**Figure 9.**
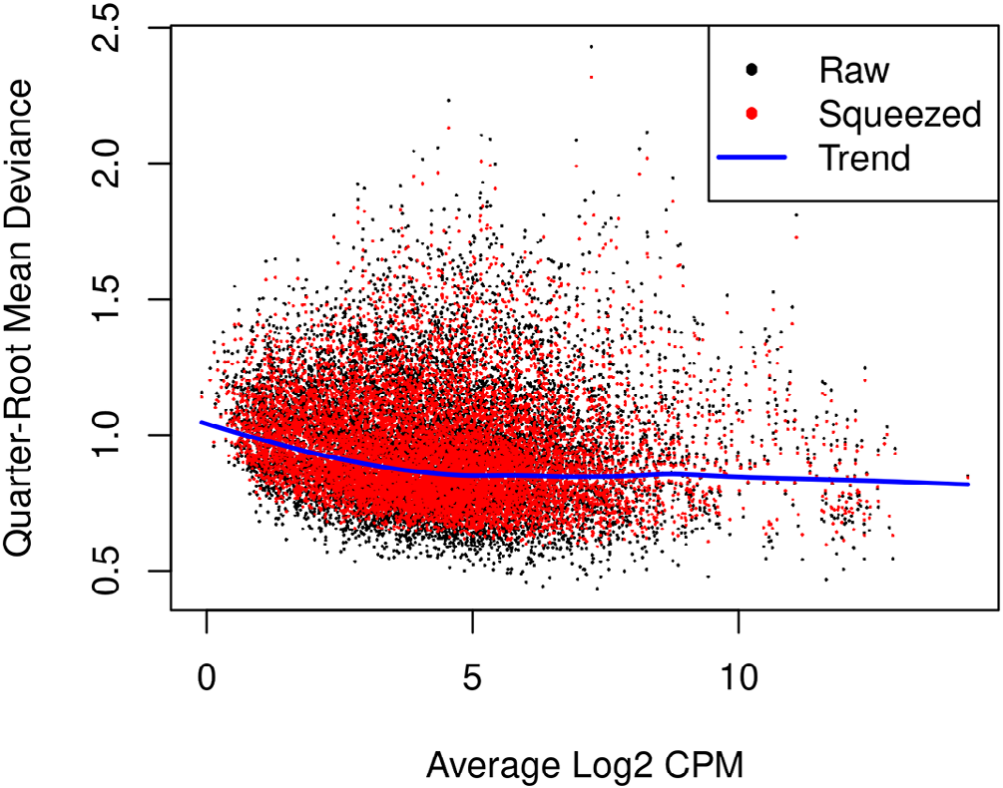
A scatter plot of the quarter-root QL dispersion against the average abundance of each gene. Estimates are shown for the raw, trended and squeezed dispersions.

The glmQLFit function is used to fit a QL model and estimate QL dispersions. The QL dispersion estimates can be visualized by plotQLDisp ( Figure 9).
> fit <- glmQLFit(y, design, robust=TRUE)
> plotQLDisp(fit)


### Time course trend analysis

The QL F-tests are performed by glmQLFTest in *edgeR* to identify genes that change significantly along the pseudotime. The tests are conducted on all three covariates of the spline model matrix. This is because the significance of any of the three coefficients would indicate a strong correlation between gene expression and pseudotime.
> res <- glmQLFTest(fit, coef=2:4)


The number of genes significantly associated with pseudotime (FDR < 0.05) are shown below.
> summary(decideTests(res))

       Z3-Z2-Z1
NotSig     8282
Sig        3268


Top significant genes can be viewed by topTags.
> topTags(res, n=10L)

Coefficient: Z1 Z2 Z3
             Ensembl_geneid   Symbol ENTREZID logFC.Z1 logFC.Z2 logFC.Z3 logCPM
Dll1     ENSMUSG00000014773     Dll1    13388   -12.55   -0.284    1.000   5.17
Tns1     ENSMUSG00000055322     Tns1    21961    -9.08    0.934   -4.569   3.77
Ptpre    ENSMUSG00000041836    Ptpre    19267    -8.04    1.849   -0.141   5.43
Kcnmb1   ENSMUSG00000020155   Kcnmb1    16533   -15.84   -0.688    2.515   2.69
Tpm2     ENSMUSG00000028464     Tpm2    22004   -12.37   -2.814    0.801   9.65
Cnn1     ENSMUSG00000001349     Cnn1    12797   -15.48   -2.363    1.571   8.53
Tacstd2  ENSMUSG00000051397  Tacstd2    56753     6.72    2.844    1.276   7.82
Marveld2 ENSMUSG00000021636 Marveld2   218518     6.04    3.077    2.532   4.64
Acta2    ENSMUSG00000035783    Acta2    11475   -15.43   -2.898    1.944  11.62
Spock2   ENSMUSG00000058297   Spock2    94214    -8.71    1.099   -3.875   2.46
            F   PValue      FDR
Dll1     77.0 8.40e-12 9.71e-08
Tns1     66.9 3.40e-11 1.96e-07
Ptpre    63.4 5.75e-11 2.21e-07
Kcnmb1   51.9 4.00e-10 1.15e-06
Tpm2     50.3 5.73e-10 1.32e-06
Cnn1     48.7 7.80e-10 1.37e-06
Tacstd2  48.0 8.32e-10 1.37e-06
Marveld2 46.6 1.11e-09 1.61e-06
Acta2    45.8 1.38e-09 1.72e-06
Spock2   44.1 1.87e-09 1.72e-06


The logFC.Z1, logFC.Z2, and logFC.Z3 values in the table above denote the estimated coefficients of Z1, Z2, and Z3 for each gene. It should be noted that these values do not carry the same interpretation as log-fold changes in traditional RNA-seq differential expression analysis. For each gene, the sign of the coefficient logFC.Z1 indicates whether the expression level of that gene increases or decreases along pseudotime in general. The top increasing and the top decreasing genes are listed below.
> tab <- topTags(res, n=Inf)**$**table
> tab**$**trend <- ifelse(tab**$**logFC.Z1 > 0, "Up", "Down")
> tab.up <- tab[tab**$**trend == "Up", ]
> tab.down <- tab[tab**$**trend == "Down", ]
> head(tab.up)

             Ensembl_geneid   Symbol ENTREZID logFC.Z1 logFC.Z2 logFC.Z3 logCPM
Tacstd2  ENSMUSG00000051397  Tacstd2    56753     6.72    2.844     1.28   7.82
Marveld2 ENSMUSG00000021636 Marveld2   218518     6.04    3.077     2.53   4.64
Tjp3     ENSMUSG00000034917     Tjp3    27375     8.26    3.173     4.97   4.50
Ehf      ENSMUSG00000012350      Ehf    13661     8.02    6.646     5.27   6.65
Cmtm8    ENSMUSG00000041012    Cmtm8    70031     8.72    0.287     1.58   6.65
Aktip    ENSMUSG00000031667    Aktip    14339     3.17    1.278     0.18   5.23
            F   PValue      FDR trend
Tacstd2  48.0 8.32e-10 1.37e-06    Up
Marveld2 46.6 1.11e-09 1.61e-06    Up
Tjp3     44.0 1.88e-09 1.72e-06    Up
Ehf      41.0 3.69e-09 2.37e-06    Up
Cmtm8    38.7 6.24e-09 2.39e-06    Up
Aktip    35.6 1.33e-08 3.50e-06    Up

> head(tab.down)

           Ensembl_geneid Symbol ENTREZID logFC.Z1 logFC.Z2 logFC.Z3 logCPM    F
Dll1   ENSMUSG00000014773   Dll1    13388   -12.55   -0.284    1.000   5.17 77.0
Tns1   ENSMUSG00000055322   Tns1    21961    -9.08    0.934   -4.569   3.77 66.9
Ptpre  ENSMUSG00000041836  Ptpre    19267    -8.04    1.849   -0.141   5.43 63.4
Kcnmb1 ENSMUSG00000020155 Kcnmb1    16533   -15.84   -0.688    2.515   2.69 51.9
Tpm2   ENSMUSG00000028464   Tpm2    22004   -12.37   -2.814    0.801   9.65 50.3
Cnn1   ENSMUSG00000001349   Cnn1    12797   -15.48   -2.363    1.571   8.53 48.7
         PValue      FDR trend
Dll1   8.40e-12 9.71e-08  Down
Tns1   3.40e-11 1.96e-07  Down
Ptpre  5.75e-11 2.21e-07  Down
Kcnmb1 4.00e-10 1.15e-06  Down
Tpm2   5.73e-10 1.32e-06  Down
Cnn1   7.80e-10 1.37e-06  Down


**Figure 10.**
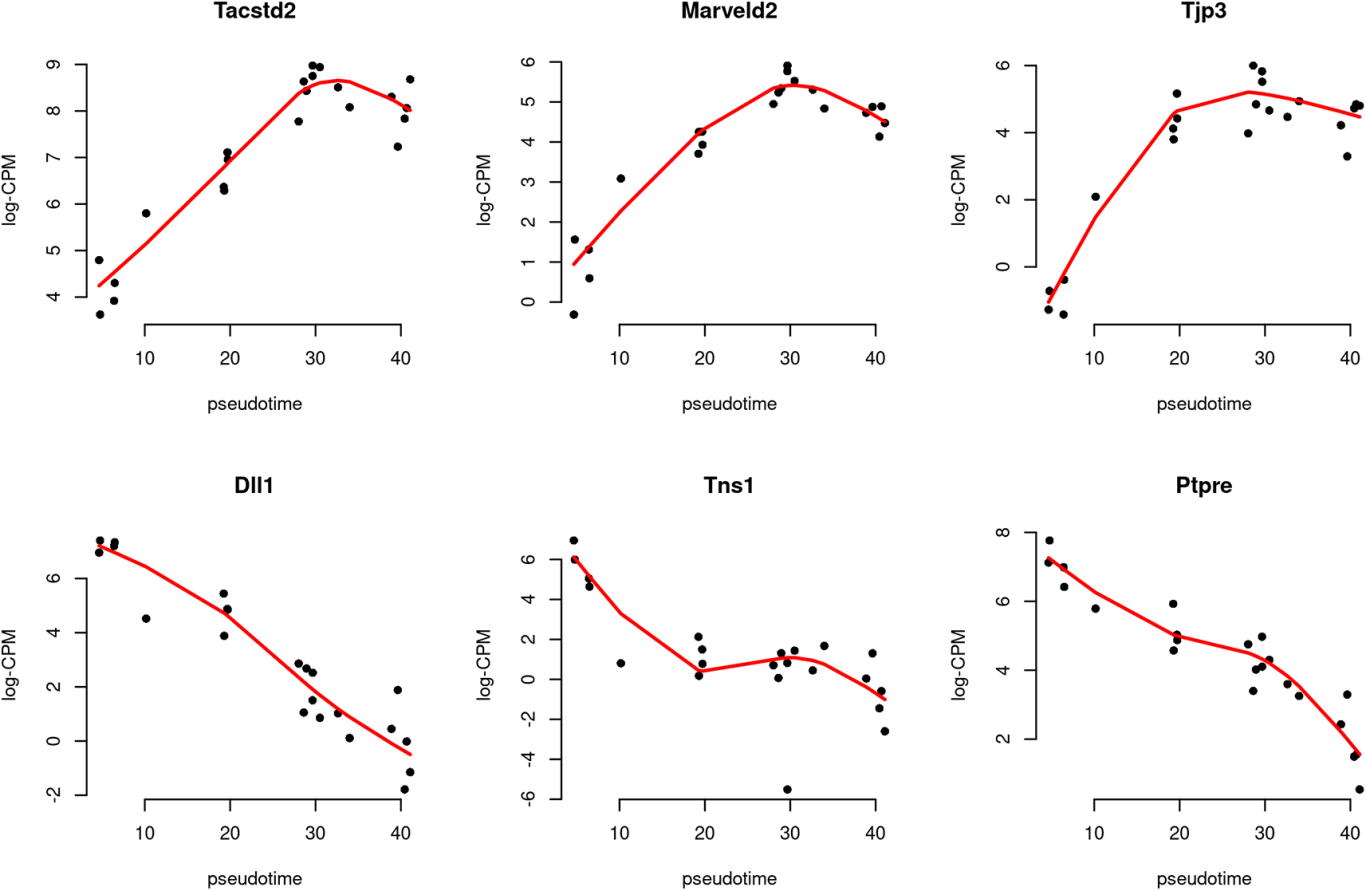
Scatter plots of expression of top genes along pseudotime. The black dots indicate the observed values, while the red line represents the fitted values calculated along pseudotime.

Scatter plots are produced to visualize the relationship between gene expression level and pseudotime for the top 3 increasing and the top 3 decreasing genes ( Figure 10). Each point in the scatter plot indicates the observed logCPM of a pseudo-bulk sample at its average pseudotime. A smooth curve is drawn along pseudotime for each gene using the fitted logCPM values obtained from the spline model. The cpm function in *edgeR* is used to calculate the observed and fitted logCPM. Since there is a cpm function in the *SingleCellExperiment* package, we use edgeR::cpm to explicitly call the cpm function in *edgeR.* The smooth curves for the top row’s 3 genes exhibit a generally increasing trend in gene expression over pseudotime, while the curves for the bottom row’s 3 genes show a general decreasing trend.
> logCPM.obs <- edgeR::cpm(y, log=TRUE, prior.count=fit**$**prior.count)
> logCPM.fit <- edgeR::cpm(fit, log=TRUE)
> topGenes <- c(rownames(tab.up)[1:3], rownames(tab.down)[1:3])
> par(mfrow=c(2,3))
> for(i in 1:6) {
+  Symbol <- topGenes[i]
+  logCPM.obs.i <- logCPM.obs[Symbol, ]
+  logCPM.fit.i <- logCPM.fit[Symbol, ]
+  plot(y**$**samples**$**pseudotime, logCPM.obs.i, xlab="pseudotime",
+     ylab="log-CPM", main=Symbol, pch=16, frame=FALSE)
+  lines(y**$**samples**$**pseudotime, logCPM.fit.i, col="red", lwd=2)
+ }


**Figure 11.**
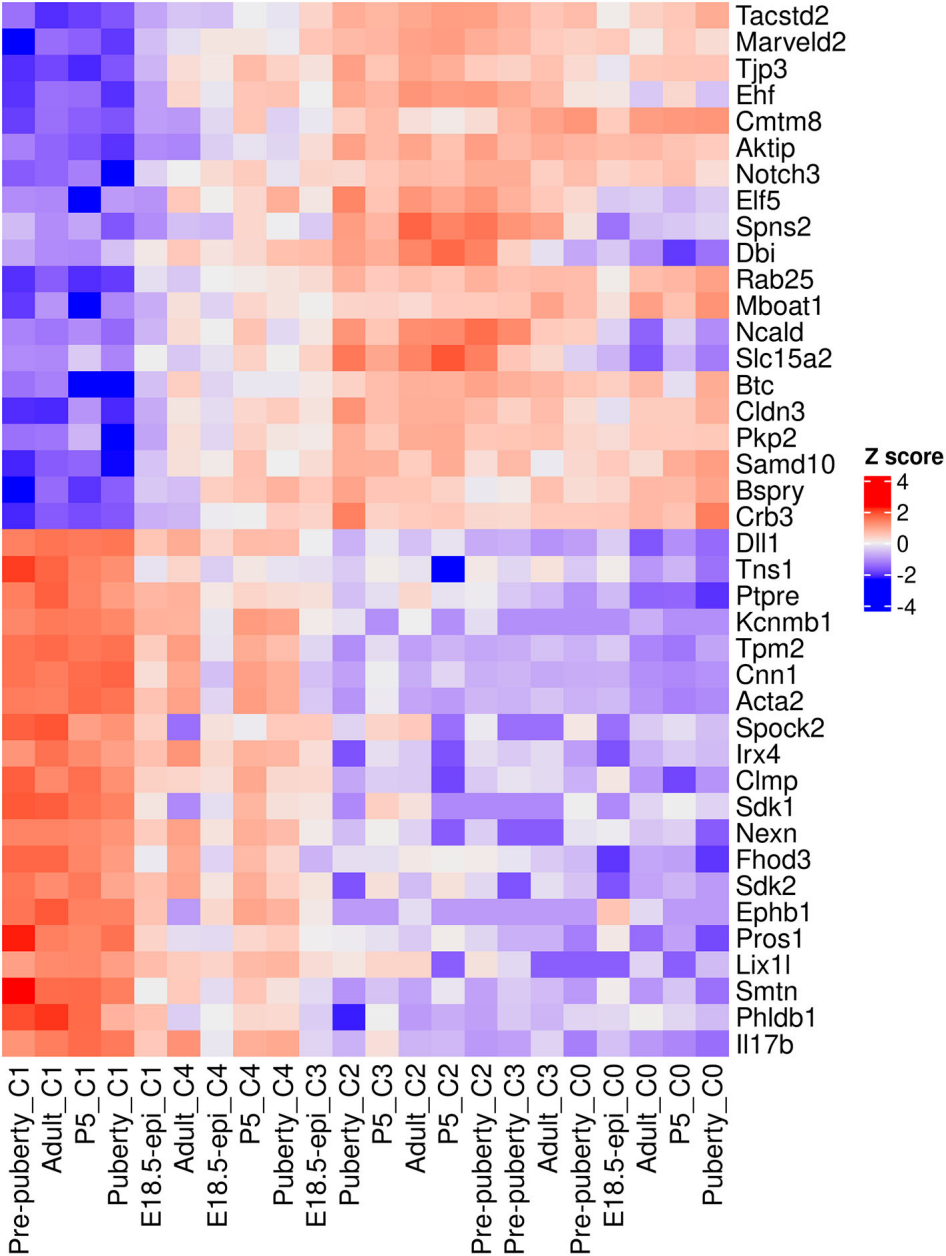
Heatmap of top 20 up and top 20 down genes. Rows are genes and columns are pseudo-bulk samples.

A heatmap is generated to examine the top 20 up and top 20 down genes collectively ( Figure 11). In the heatmap, pseudo-bulk samples are arranged in increasing pseudotime from left to right. The up genes are on the top half of the heatmap whereas the down genes are on the bottom half. The heatmap shows a gradual increase in expression levels of the up genes from left to right, while the down genes display the opposite trend.
> topGenes <- c(rownames(tab.up)[1:20], rownames(tab.down)[1:20])
> z <- logCPM.obs[topGenes, ]
> z <- t(scale(t(z)))
> ComplexHeatmap::Heatmap(z, name = "Z score",
+   cluster_rows = FALSE,cluster_columns = FALSE)


## Time course functional enrichment analysis

### Gene ontology analysis

To interpret the results of the time course analysis at the functional level, we perform gene set enrichment analysis. Gene ontology (GO) is one of the commonly used databases for this purpose. The GO terms in the GO databases are categorized into three classes: biological process (BP), cellular component (CC) and molecular function (MF). In a GO analysis, we are interested in finding GO terms that are over-represented or enriched with significant genes.

GO analysis is usually directional. For simplicity, we re-perform the QL F-test on the Z1 coefficient to identify genes that exhibit a general linear increase or decrease along pseudotime. The numbers of genes with a significant increasing or decreasing linear trend are shown below.
> res_2 <- glmQLFTest(fit, coef=2)
> summary(decideTests(res_2))

         Z1
Down   1366
NotSig 9056
Up     1128


To perform a GO analysis, we apply the goana function to the above test results. Note that Entrez gene IDs are required for goana, which has been added to the ENTREZID column in the gene annotation. The top enriched GO terms can be viewed using topGO function.
> go <- goana(res_2, geneid="ENTREZID", species="Mm")
> topGO(go, truncate.term = 30, n=15)

                                     Term Ont    N  Up Down     P.Up   P.Down
GO:0071944                 cell periphery  CC 2928 414  629 3.59e-19 1.60e-70
GO:0005576           extracellular region  CC  995 133  279 7.51e-05 7.77e-49
GO:0030312 external encapsulating stru...  CC  297  33  134 2.40e-01 3.56e-48
GO:0031012           extracellular matrix  CC  297  33  134 2.40e-01 3.56e-48
GO:0009653 anatomical structure morpho...  BP 1828 178  413 5.32e-01 6.42e-47
GO:0005886                plasma membrane  CC 2650 380  519 2.58e-18 4.70e-41
GO:0005615            extracellular space  CC  674 100  204 1.06e-05 1.95e-40
GO:0048731             system development  BP 2741 261  528 7.01e-01 1.04e-39
GO:0062023 collagen-containing extrace...  CC  240  28  109 1.84e-01 1.48e-39
GO:0007155                  cell adhesion  BP  833 107  228 1.61e-03 1.59e-37
GO:0048856 anatomical structure develo...  BP 3657 351  643 6.72e-01 5.30e-37
GO:0032501 multicellular organismal pr...  BP 4101 417  699 1.48e-01 1.01e-36
GO:0007275 multicellular organism deve...  BP 3188 295  577 8.82e-01 1.26e-35
GO:0032502          developmental process  BP 3917 382  671 5.26e-01 3.02e-35
GO:0048513       animal organ development  BP 2172 210  432 5.81e-01 2.85e-34


**Figure 12.**
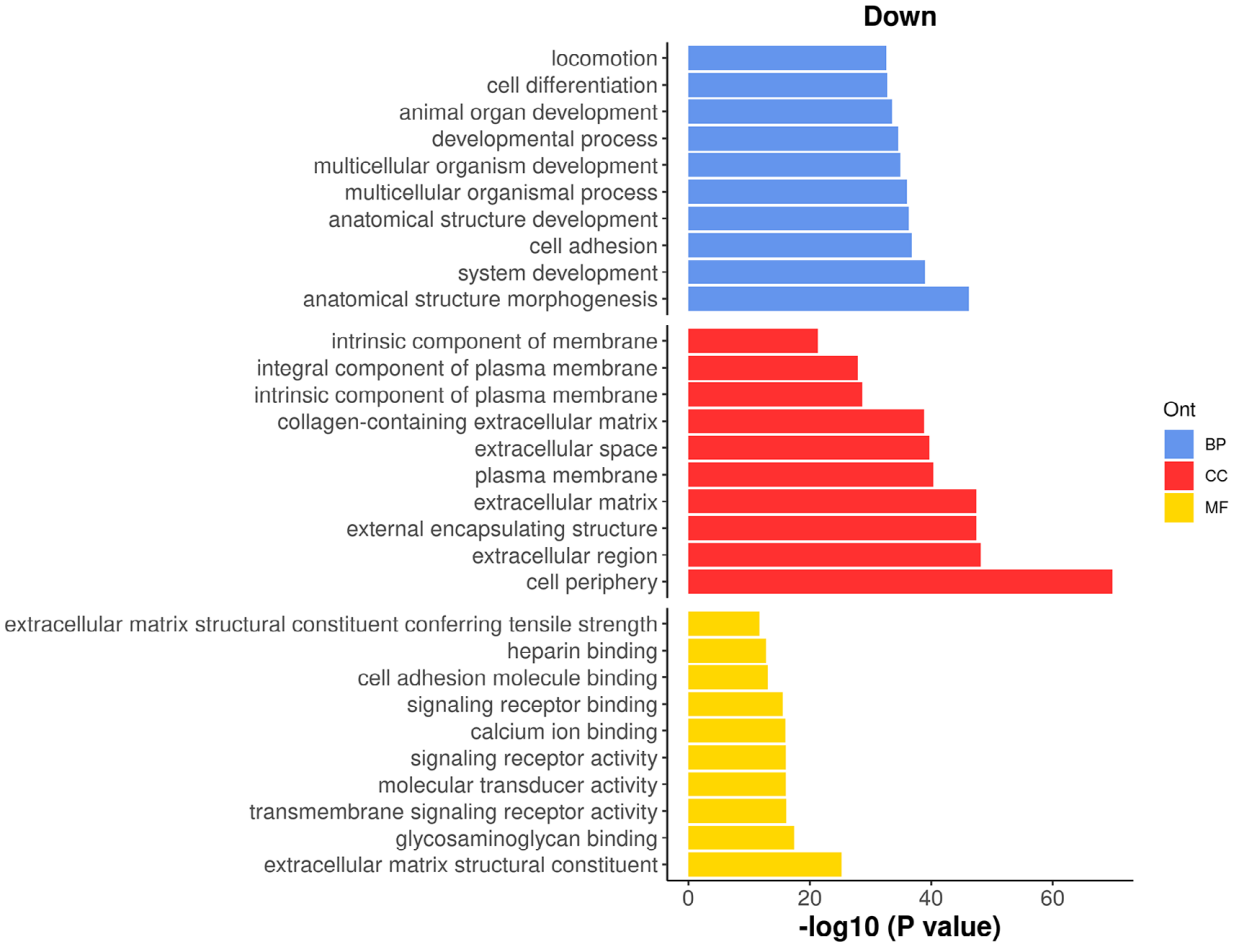
Barplot of $-\log_{10}$ p-values of the top 10 down-regulated GO terms under each GO category.

It can be seen that most of the top GO terms are down-regulated. Here, we choose the top 10 down-regulated terms for each GO category and show the results in a barplot ( Figure 12).
> top_go <- rbind.data.frame(topGO(go, ont =c("BP"), sort="Down",n=10),
+                            topGO(go, ont =c("CC"), sort="Down",n=10),
+                            topGO(go, ont =c("MF"), sort="Down",n=10))
> d <- transform(top_go, P_DE = P.Down, neg_log10_P = -log10(P.Down))
> d**$**Term <- factor(d**$**Term,levels = d**$**Term)
> ggplot(data = d, aes(x = neg_log10_P, y = Term, fill = Ont) ) +
+  geom_bar(stat = "identity", show.legend = TRUE) +
+  labs(x="-log10 (P value)", y="", title = "Down") +
+  facet_grid(Ont~.,scales = "free",space = "free") +
+  my_theme_ggplot + my_theme_facet +
+  scale_fill_manual(values = my_colors_15[-2]) +
+  theme(strip.text = ggplot2::element_blank())


### KEGG pathway analysis

The Kyoto Encyclopedia of Genes and Genomes ^23^ (KEGG) is another commonly used database for exploring signaling pathways to understand the molecular mechanism of diseases and biological processes. A KEGG analysis can be done by using kegga function.

The top enriched KEGG pathways can be viewed by using topKEGG function.
> kegg <- kegga(res_2, geneid="ENTREZID", species="Mm")
> topKEGG(kegg, truncate.path=40, n=15)

                                          Pathway   N Up Down     P.Up   P.Down
mmu04512                 ECM-receptor interaction  56  4   30 8.10e-01 3.49e-14
mmu04510                           Focal adhesion 157 12   52 8.51e-01 1.28e-12
mmu04974         Protein digestion and absorption  49  6   26 3.44e-01 2.42e-12
mmu05414                   Dilated cardiomyopathy  53  1   21 9.96e-01 2.18e-07
mmu04015                   Rap1 signaling pathway 150  9   40 9.63e-01 4.43e-07
mmu04270       Vascular smooth muscle contraction  79  6   26 7.96e-01 6.22e-07
mmu05412 Arrhythmogenic right ventricular card...  44  3   18 8.17e-01 9.15e-07
mmu04020                Calcium signaling pathway 108 11   31 4.89e-01 1.59e-06
mmu05410              Hypertrophic cardiomyopathy  52  3   19 8.95e-01 3.48e-06
mmu04530                           Tight junction 129 30   27 4.92e-06 2.09e-03
mmu04024                   cAMP signaling pathway 119 12   32 4.99e-01 5.08e-06
mmu04151               PI3K-Akt signaling pathway 242 20   53 8.16e-01 5.32e-06
mmu04921               Oxytocin signaling pathway  93  5   27 9.56e-01 5.83e-06
mmu04360                            Axon guidance 143 11   36 8.37e-01 6.97e-06
mmu00532 Glycosaminoglycan biosynthesis - chon...  15  1    9 7.86e-01 1.13e-05


**Figure 13.**
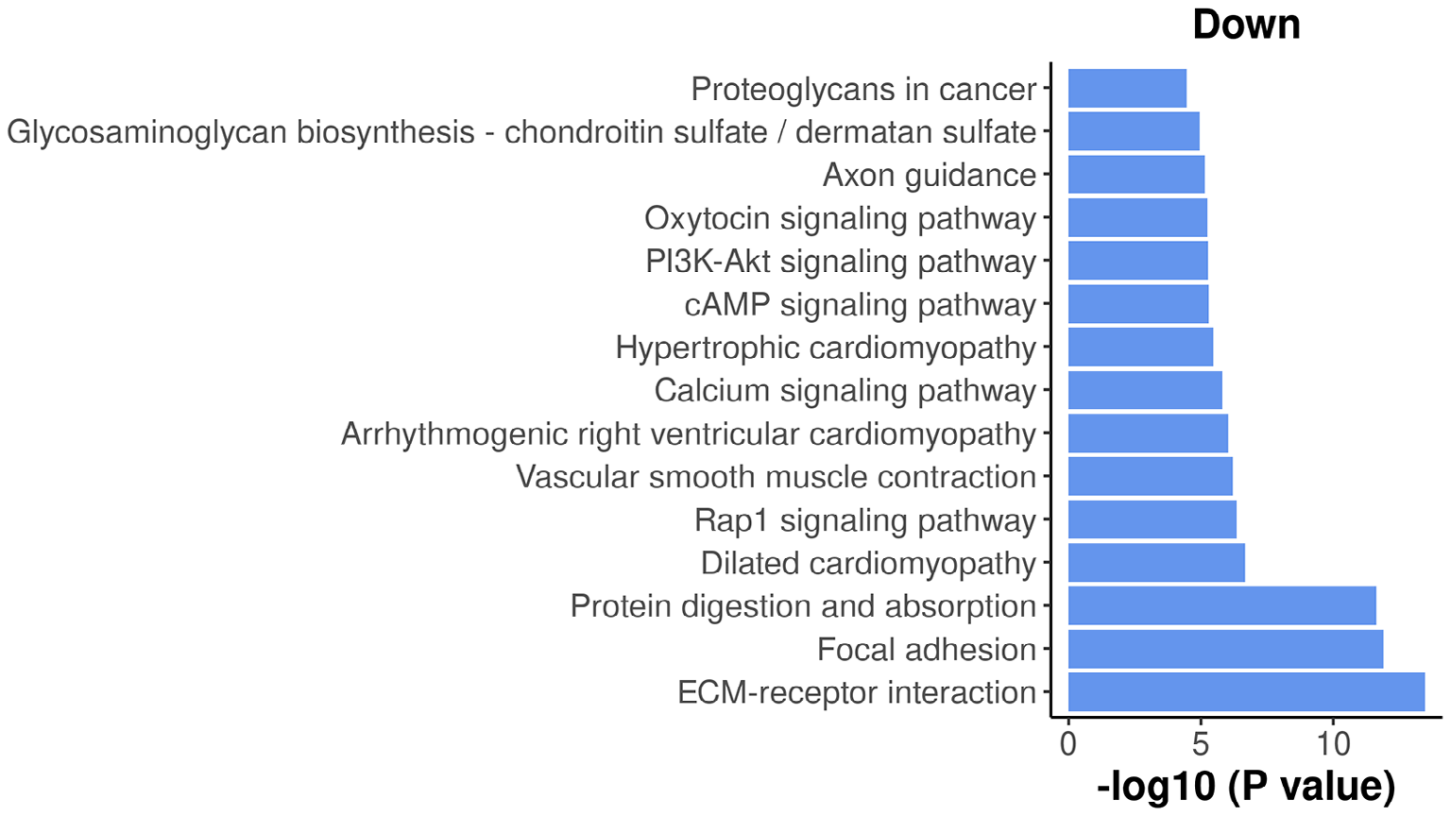
Bar plot of $-\log_{10}$ p-values of the top 15 down-regulated KEGG pathways.

The results show that most of the top enriched KEGG pathways are down-regulated. Here, we select the top 15 down-regulated KEGG pathways and visualize their significance in a bar plot ( Figure 13).
> top_path <- topKEGG(kegg,sort="Down",n=15)
> data_for_barplot <- transform(top_path, P_DE=P.Down, neg_log10_P=-log10(P.Down))
> data_for_barplot**$**Pathway <- factor(data_for_barplot**$**Pathway,
+                                    levels=data_for_barplot**$**Pathway)
> ggplot(data=data_for_barplot,aes(x=neg_log10_P, y=Pathway) ) +
+   geom_bar(stat="identity", show.legend=FALSE, fill=my_colors_15[1]) +
+   labs(x="-log10 (P value)", y="", title="Down" ) +
+   my_theme_ggplot


Among the top down-regulated pathways, the PI3K-Akt signaling pathway is noteworthy as it is typically involved in cell proliferation and plays a crucial role in mammary gland development.

To assess the overall expression level of the PI3K-Akt signaling pathway across pseudotime, a plot is generated by plotting the average expression level of all the genes in the pathway against pseudotime. The information of all the genes in the pathway can be obtained by getGeneKEGGLinks and getKEGGPathwayNames.
> kegg_links <- getGeneKEGGLinks("mmu")
> p_names <- getKEGGPathwayNames("mmu")
> p1 <- p_names[grep("PI3K", p_names**$**Description), ]
> p1_GeneIDs <- subset(kegg_links, PathwayID == p1**$**PathwayID)**$**GeneID
> tab_p1 <- tab[tab**$**ENTREZID %in% p1_GeneIDs, ]
> d <- logCPM.obs[tab_p1**$**Symbol,]
> d <- apply(d, 2, mean)
> d <- data.frame(avg_logCPM = d, avg_pseudotime = y**$**samples**$**pseudotime)
> head(d)

               avg_logCPM avg_pseudotime
Pre-puberty_C1       4.44           4.65
Adult_C1             4.67           4.77
P5_C1                4.70           6.41
Puberty_C1           4.17           6.48
E18.5-epi_C1         4.45          10.16
Adult_C4             3.51          19.25


**Figure 14.**
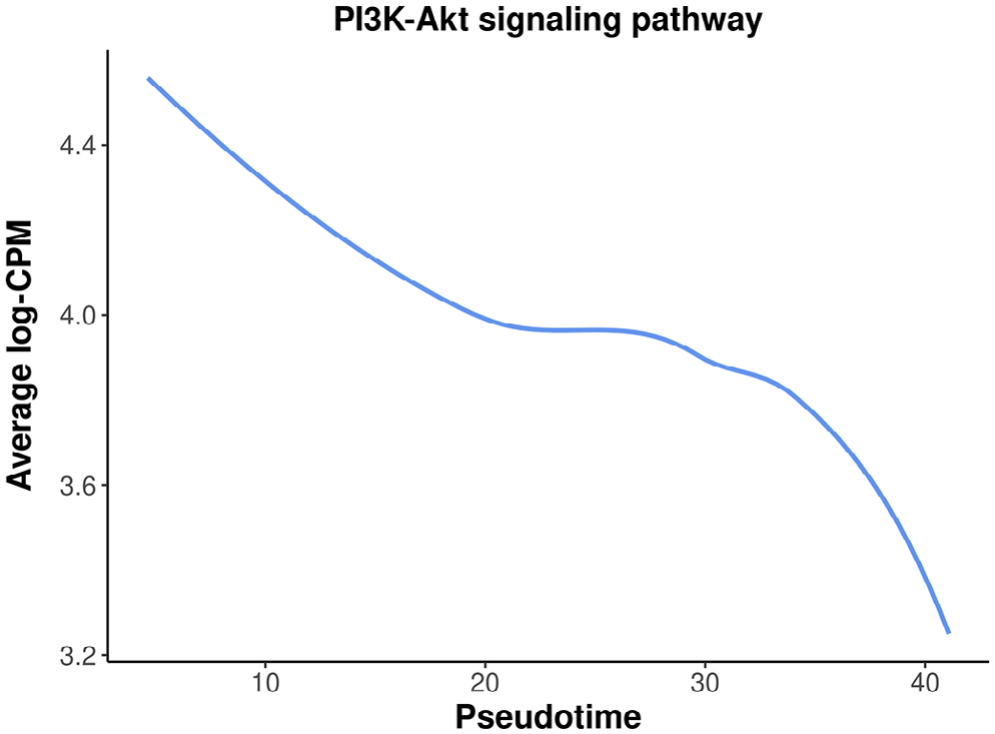
A smooth curve of PI3K-Akt signaling pathway expression level against pseudotime.

The plot below clearly illustrates a significant down-regulation of the PI3K-Akt pathway along pseudotime ( Figure 14).
> ggplot(data = d,aes(x = avg_pseudotime, y = avg_logCPM) ) +
+   geom_smooth(color=my_colors_15[1],se = FALSE) +
+   labs(x="Pseudotime", y="Average log-CPM",
+      title = "PI3K-Akt signaling pathway" ) +
+   my_theme_ggplot


## Discussion

In this article, we demonstrated a complete workflow of a pseudo-temporal trajectory analysis of scRNA-seq data. This workflow takes single-cell count matrices as input and leverages the Seurat pipeline for standard scRNA-seq analysis, including quality control, normalization, and integration. The *scDblFinder* package is utilized for doublet prediction. Trajectory inference is conducted with *monocle3*, while the *edgeR* QL framework with a pseudo-bulking strategy is applied for pseudo-time course analysis. Alternative methods and packages can be used interchangeably with the ones implemented in this study, as long as they perform equivalent functions. For instance, the bioconductor workflow may be substituted for the Seurat pipeline in scRNA-seq analysis, whereas the *slingshot* package may replace *monocle3* for performing trajectory analysis.

This workflow article utilized 10x scRNA-seq data from five distinct stages of mouse mammary gland development, with a focus on the lineage progression of epithelial cells. By performing a time course analysis based on pseudotime along the developmental trajectory, we successfully identified genes and pathways that exhibit differential expression patterns over the course of pseudotime. The results of this extensive analysis not only confirm previous findings in the literature regarding the mouse mammary gland epithelium, but also reveal new insights specific to the early developmental stages of the mammary gland. The analytical framework presented here can be utilized for any single-cell experiments aimed at studying dynamic changes along a specific path, whether it involves cell differentiation or the development of cell types.

## Packages used

This workflow depends on various packages from the Bioconductor project version 3.15 and the Comprehensive R Archive Network (CRAN), running on R version 4.2.1 or higher. The complete list of the packages used for this workflow are shown below:
> sessionInfo()

R version 4.2.1 (2022-06-23)
Platform: x86_64-pc-linux-gnu (64-bit)
Running under: CentOS Linux 7 (Core)

Matrix products: default
BLAS:   /stornext/System/data/apps/R/R-4.2.1/lib64/R/lib/libRblas.so
LAPACK: /stornext/System/data/apps/R/R-4.2.1/lib64/R/lib/libRlapack.so

locale:
 [1] LC_CTYPE=en_AU.UTF-8       LC_NUMERIC=C
 [3] LC_TIME=en_AU.UTF-8        LC_COLLATE=en_AU.UTF-8
 [5] LC_MONETARY=en_AU.UTF-8    LC_MESSAGES=en_AU.UTF-8
 [7] LC_PAPER=en_AU.UTF-8       LC_NAME=C
 [9] LC_ADDRESS=C               LC_TELEPHONE=C
[11] LC_MEASUREMENT=en_AU.UTF-8 LC_IDENTIFICATION=C

attached base packages:
 [1] stats4  stats   graphics  grDevices utils  datasets methods  base

other attached packages:
 [1] org.Mm.eg.db_3.15.0         AnnotationDbi_1.58.0
 [3] monocle3_1.2.9              SingleCellExperiment_1.18.0
 [5] SummarizedExperiment_1.26.1 GenomicRanges_1.48.0
 [7] GenomeInfoDb_1.32.2         IRanges_2.30.0
 [9] S4Vectors_0.34.0            MatrixGenerics_1.8.0
[11] matrixStats_0.62.0          Biobase_2.56.0
[13] BiocGenerics_0.42.0         scDblFinder_1.10.0
[15] sp_1.5-0                    SeuratObject_4.1.0
[17] Seurat_4.1.1                ggplot2_3.3.6
[19] edgeR_3.38.1                limma_3.55.5

loaded via a namespace (and not attached):
 [1] utf8_1.2.2              R.utils_2.11.0             reticulate_1.25
 [4] lme4_1.1-29             tidyselect_1.1.2           RSQLite_2.2.14
 [7] htmlwidgets_1.5.4       grid_4.2.1                 BiocParallel_1.30.3
[10] Rtsne_0.16              munsell_0.5.0              ScaledMatrix_1.4.0
[13] codetools_0.2-18        ica_1.0-2                  statmod_1.4.36
[16] scran_1.24.0            xgboost_1.6.0.1            future_1.26.1
[19] miniUI_0.1.1.1          withr_2.5.0                spatstat.random_2.2-0
[22] colorspace_2.0-3        progressr_0.10.1           highr_0.9
[25] knitr_1.39              ROCR_1.0-11                tensor_1.5
[28] listenv_0.8.0           labeling_0.4.2             GenomeInfoDbData_1.2.8
[31] polyclip_1.10-0         bit64_4.0.5                farver_2.1.0
[34] parallelly_1.32.0       vctrs_0.4.1                generics_0.1.2
[37] xfun_0.31               doParallel_1.0.17          R6_2.5.1
[40] clue_0.3-61             ggbeeswarm_0.6.0           rsvd_1.0.5
[43] locfit_1.5-9.5          cachem_1.0.6               bitops_1.0-7
[46] spatstat.utils_2.3-1    DelayedArray_0.22.0        assertthat_0.2.1
[49] promises_1.2.0.1        BiocIO_1.6.0               scales_1.2.0
[52] rgeos_0.5-9             beeswarm_0.4.0             gtable_0.3.0
[55] beachmat_2.12.0         Cairo_1.5-15               globals_0.15.0
[58] goftest_1.2-3           rlang_1.0.2                GlobalOptions_0.1.2
[61] splines_4.2.1           rtracklayer_1.56.0         lazyeval_0.2.2
[64] spatstat.geom_2.4-0     BiocManager_1.30.18        yaml_2.3.5
[67] reshape2_1.4.4          abind_1.4-5                httpuv_1.6.5
[70] tools_4.2.1             ellipsis_0.3.2             spatstat.core_2.4-4
[73] RColorBrewer_1.1-3      proxy_0.4-27               ggridges_0.5.3
[76] Rcpp_1.0.8.3            plyr_1.8.7                 sparseMatrixStats_1.8.0
[79] zlibbioc_1.42.0         purrr_0.3.4                RCurl_1.98-1.7
[82] rpart_4.1.16            deldir_1.0-6               GetoptLong_1.0.5
[85] pbapply_1.5-0           viridis_0.6.2              cowplot_1.1.1
[88] zoo_1.8-10              ggrepel_0.9.1              cluster_2.1.3
[91] magrittr_2.0.3          data.table_1.14.2          RSpectra_0.16-1
[94] scattermore_0.8         circlize_0.4.15            lmtest_0.9-40
[97] RANN_2.6.1              fitdistrplus_1.1-8         patchwork_1.1.1
[100] mime_0.12              evaluate_0.15              xtable_1.8-4
[103] XML_3.99-0.10          shape_1.4.6                gridExtra_2.3
[106] compiler_4.2.1         scater_1.24.0              tibble_3.1.7
[109] KernSmooth_2.23-20     crayon_1.5.1               R.oo_1.25.0
[112] minqa_1.2.4            htmltools_0.5.2            mgcv_1.8-40
[115] later_1.3.0            tidyr_1.2.0                DBI_1.1.3
[118] ComplexHeatmap_2.12.0  MASS_7.3-57                boot_1.3-28
[121] leidenbase_0.1.11      Matrix_1.5-3               cli_3.3.0
[124] R.methodsS3_1.8.2      parallel_4.2.1             metapod_1.4.0
[127] igraph_1.3.2           pkgconfig_2.0.3            GenomicAlignments_1.32.0
[130] terra_1.5-34           plotly_4.10.0              scuttle_1.6.2
[133] spatstat.sparse_2.1-1  foreach_1.5.2              vipor_0.4.5
[136] dqrng_0.3.0            XVector_0.36.0             stringr_1.4.0
[139] digest_0.6.29          sctransform_0.3.3          RcppAnnoy_0.0.19
[142] spatstat.data_2.2-0    Biostrings_2.64.0          leiden_0.4.2
[145] uwot_0.1.11            DelayedMatrixStats_1.18.0  restfulr_0.0.15
[148] shiny_1.7.1            Rsamtools_2.12.0           nloptr_2.0.3
[151] rjson_0.2.21           lifecycle_1.0.1            nlme_3.1-158
[154] jsonlite_1.8.0         SeuratWrappers_0.3.0       BiocNeighbors_1.14.0
[157] viridisLite_0.4.0      fansi_1.0.3                pillar_1.7.0
[160] lattice_0.20-45        GO.db_3.15.0               KEGGREST_1.36.2
[163] fastmap_1.1.0          httr_1.4.3                 survival_3.3-1
[166] remotes_2.4.2          glue_1.6.2                 iterators_1.0.14
[169] png_0.1-7              bit_4.0.4                  bluster_1.6.0
[172] stringi_1.7.6          blob_1.2.3                 BiocSingular_1.12.0
[175] memoise_2.0.1          dplyr_1.0.9                irlba_2.3.5
[178] future.apply_1.9.0


## Data Availability

The single-cell RNA-seq datasets used in this study were obtained from the Gene Expression Omnibus (GEO) with accession numbers of GSE103275 ^16^ and GSE164017. ^17^

## Software availability

Source code available from: https://github.com/jinming-cheng/TimeCoursePaperWorkflow

Archived source code at time of publication: https://doi.org/10.5281/zenodo.7879833 ^24^

License: GNU General Public License version 3 (GPL-3.0-only)

All the packages used in this workflow are publicly available from the Bioconductor project (version 3.15) and the Comprehensive R Archive Network ( CRAN).
