# Supplementary material for: Unraveling the timeline of gene expression: A pseudotemporal trajectory analysis of single-cell RNA sequencing data
Source: F1000Res. 2023 Nov 10;12:684. Originally published 2023 Jun 15. [Version 2] doi: 10.12688/f1000research.134078.2 (PMC10663991; doi:10.12688/f1000research.134078.2)
Supplement: Peer review history

# Reviewer report: Reviewer response for version 2

**DOI:** 10.5256/f1000research.157785.r222365

**Published:** 2024-11-07

**Authors:** Koen Van den berge

**Affiliations:** 1 Statistics and Decision Sciences, Janssen R&D, Beerse, Belgium

Thank you to the authors for carefully responding to the comments provided, and my apologies for a belated response. I have one main point remaining, which is on the pseudobulking strategy adopted in the paper. Notwithstanding my approval of the manuscript in its current form, evaluating the suggestion could still be a useful exercise.

 While I agree with the authors that pseudobulking the traditional way, i.e., by cluster x sample, is the most straight-forward, this does not mean that it is the best way for the specific problem. One could also construct bins of pseudotime, e.g., 5 bins just like 5 clusters were identified, and aggregate counts by pseudotime bin x sample. This seems like a more relevant grouping for the problem at hand, and avoids the awkward pseudotime distributions mentioned in my first review ( https://github.com/jinming-cheng/TimeCoursePaperWorkflow/issues/1).

 Finally, I also thank Professor Smyth’s additional text on regression versus smoothing splines. It may be worthwhile to summarize these points as part of the paper to motivate the choice for regression splines.

Is the rationale for developing the new method (or application) clearly explained?

Yes

Is the description of the method technically sound?

Partly

Are the conclusions about the method and its performance adequately supported by the findings presented in the article?

Yes

If any results are presented, are all the source data underlying the results available to ensure full reproducibility?

Yes

Are sufficient details provided to allow replication of the method development and its use by others?

Yes

Reviewer Expertise:

Statistical omics

I confirm that I have read this submission and believe that I have an appropriate level of expertise to confirm that it is of an acceptable scientific standard.

# Reviewer report: Reviewer response for version 2

**DOI:** 10.5256/f1000research.157785.r222366

**Published:** 2023-11-20

**Authors:** Michael D Morgan

**Affiliations:** 1 Institute of Medical Sciences, School of Medicine, Medical Sciences and Nutrition, University of Aberdeen, Aberdeen, Scotland, UK

The authors have largely addressed my previous concerns with the manuscript. A few minor issues are outstanding however. 

 In their response to my comments, and in the revised manuscript, the authors state "In general, the same cellranger reference build is preferred for consistency, although the effect on the downstream analysis is negligible." This trivialises the importance of harmonised genome/transcriptome annotations. While the impact on *this* analysis may be negligible, that may not hold across all data and analyses. The authors should note that it does not impact on *their specific analysis*. The reason for this nit-picking is so as not to instill bad habits in newcomers to the field.

 In response to a comment on the linear vs. bifurcating nature of the trajectory. It would be useful for readers to understand that this particular epithelial lineage is known to progress from Basal -> LP -> ML. The current manuscript text can be interpreted as either ML <- Basal -> LP or Basal -> LP -> ML. This is important because the biology drives an analytical choice here - this is purely for clarity.

Is the rationale for developing the new method (or application) clearly explained?

Partly

Is the description of the method technically sound?

Partly

Are the conclusions about the method and its performance adequately supported by the findings presented in the article?

Partly

If any results are presented, are all the source data underlying the results available to ensure full reproducibility?

Yes

Are sufficient details provided to allow replication of the method development and its use by others?

Partly

Reviewer Expertise:

Computational biology, single-cell, genetics, immunology

I confirm that I have read this submission and believe that I have an appropriate level of expertise to confirm that it is of an acceptable scientific standard.

# Reviewer report: Reviewer response for version 1

**DOI:** 10.5256/f1000research.147104.r190769

**Published:** 2023-08-31

**Authors:** Anna Alemany, Xuan Quy Nguyen, Noëlle Dommann

**Affiliations:** 1 Department of Anatomy and Embryology, Leiden University Medical Center, Leiden, Netherlands Antilles

In this manuscript, Cheng *et al* describe an R pipeline to perform scRNA-seq analysis, filter out specific cell types present in different datasets, integrate them together and perform pseudo-temporal analysis using monocle3. In addition, the authors then introduce a modified RNAseq analysis to identify genes with differential expression patterns along pseudo-temporal trajectories and perform both GO and KEGG analysis.

 The pipeline that they present has the potential to become a tutorial for researchers that are starting scRNAseq analysis. However, some parts of the text and the code require extra clarification (e.g. the pseudo-bulk analysis and the fitting of dispersion parameters suffers a lot from lack of explanations). Below, we summarise some comments that we hope the authors can use to make their manuscript stronger.

- When locally rerunning the code, outcomes are not fully reproducible. Most likely this is due to the fact the authors do not set random state for the code. The authors should consider fixing this.
- Some lines of code present in the manuscript are missing on the Github repository. Is there any rational for this?
- In the introduction, python-specific pipelines (e.g. scanpy) should also be referred to.
- When introducing the pseudo-bulk method to identify marker genes (3 ^rd^ paragraph in the introduction), the authors mention that it has superior computational efficiency. Could the authors specify what they mean?
- In the last paragraph of the introduction, the authors say that they present a “new” single cell workflow. In what sense this pipeline is novel?
- In “reading the data” section, two for loops of the first gray box run from 1:5. However, to make it as general as possible for future users of the pipeline, 5 should be replace by length(samples) or length(targets).
- In “reading the data” section, the step in which a dge_merged object is created is unnecessary. All the QC analysis presented later is done at the level of individual samples: working with the elements in the list dge_all would simplify several lines of code.
- In “reading the data” section, in the last gray box the authors show number of genes. Maybe they could consider printing out also the number of reads per sample, since this is a common QC.

- In the sub-section “Quality control”, the authors could elaborate a bit more on what each QC parameter is telling us. For example, high mitochondrial genes indicate damaged or dead cells.
- In sub-section “Quality control”, the choice of thresholds (<500 number of genes, >10% mitochondria, thresholds for high number of genes) seem very arbitrary. The plots on Figure 1 should be modified to visualize better the effect of the different thresholds. In addition, a histogram of the log-number of reads per cell should be included as a QC. In addition, the y label in Figure 1 should be replaced: library size is not the same as number of counts per cell. In the caption, one should explicitly mention that each dot is a cell barcode.
- When each dataset is converted into a Seurat object, the parameters min.cells = 3 and min.features = 200 should be discussed. How relevant is the min.features parameter given the previous 500 threshold?

- In the “Standard Seurat analysis of individual sample” sub-sections, the authors should replace “the data of each sample” by the “raw counts of each sample” when describing the normalization step.
- In the “Standard Seurat analysis of individual sample” sub-section, 30 PCs are used by default to perform the umap embedding. The authors should discuss why 30, and whether/why this is a good choice for all the libraries.
- In addition, why do they use different resolution for each dataset for the cell clustering? What criteria is followed to decide on this? Maybe some Silhouette plot or Gap Statistics should be included?

- For the sake of readability, the authors could consider including a sub-section header after the first gray box of the “removing potential doublets and non-epithelial cells” sub-section.
- The choice of clusters expressing Epcam seems arbitrary: why is cluster 0 included in pre-puberty, why are not all the clusters included in puberty, and why in adult cluster 4 is not included? To select Epcam+ clusters a bit more quantitatively, a boxplot (or violin plot or heatmap) should be made displaying mean Epcam expression per cluster.
- The Expcam expression range use to select epithelial cells should either be the same for the 5 samples or follow some trend as a function of time. Can the authors show this in a plot?
- Did the authors try to re-cluster data after doublet removal? This might be impactful in some datasets (such as the Puberty one).

- In “integrating epithelial cells of five samples”, why do we need to integrate the data using the anchor-based method? How does the UMAP of all combined  epithelial cells look like without the anchor-based strategy?
- The author listed some batch-correction methods in the introduction, but here they do not explain the choice of this anchor-based method. Could they comment on this?
- Why do the authors use the min.cells = 3 parameter in the first gray box of the “integrating epithelial cells of five samples”?
- Which criteria does the “findIntegrationAnchors”to select features?
- As before, 30 PC are used. Additionally, clustering is done using a resolution of 0.2. How did the authors decide of these values?
- While rerunning the code to generate Fig. 3, a slightly different version of the UMAP was obtained. This leads to define different cell clusters and generate list of markers gene for downstream. Maybe it would be good to set a random seed in the umap or FindNeighbors to make the results from this pipeline robust.

- In “cell type identification”, the authors should consider extending the gene marker list. Why were specifically these markers selected? Csn3 and  Elf5 are markers for luminal alveolar cells that are particularly active during lactation and not in the progenitor stage. Also Prlr is considered to be involved in milk production. What about performing Gene Ontology on the clusters?
- Maybe cluster 5 should be removed from downstream analysis since it is stromal cells contamination?

- What is the definition of a “key point” along a trajectory?
- How does pseudotime estimation compare to the embryo stage of origin? A scatter plot would be very informative.

- In section “constructing pseudo-bulk profiles”, the authors aggregate cells with the same combination of sample and cluster. However, this goes against the anchor-based integration method. Could the authors discuss why they do not use pseudotime intervals to aggregate cells with similar pseudotime values?
- In the “design matrix” sub-section, could the authors discuss the structure of the design parameter? What are Z1, Z2 and Z3?
- What does CPM stand for in Figures 8 and 9?
- It would be beneficial to explain better what is the y-axis of Figure 9. This section is in general very challenging to follow for anyone that does not have experience in bulk differential expression analysis. At least, a proper definition of “dispersion” should be given.
- In Figure 10, it would be useful to color the black dots according to sample of origin to better understand the trends.
- Some of the genes shown in Figure 10 should also be displayed in the UMAP obtained with integration of epithelial cells to appreciate better gene expression patterns along pseudo-time and along cell types.

- In the Gene ontology and KEGG analysis subsections, it is not clear whether the GO is performed on the genes that exhibit a significant linear increase or decrease with pseudotime. Could the authors clarify this?

- In the discussion, the authors mention that their analysis revealed new insights specific to early developmental stages of the mammary gland. Could the authors elaborate on those?

- We would advise the authors to load all the required libraries at the beginning of the pipeline.

Is the rationale for developing the new method (or application) clearly explained?

No

Is the description of the method technically sound?

Partly

Are the conclusions about the method and its performance adequately supported by the findings presented in the article?

Partly

If any results are presented, are all the source data underlying the results available to ensure full reproducibility?

Yes

Are sufficient details provided to allow replication of the method development and its use by others?

Partly

Reviewer Expertise:

Stem cell biology; bioinformatics

We confirm that we have read this submission and believe that we have an appropriate level of expertise to confirm that it is of an acceptable scientific standard, however we have significant reservations, as outlined above.

# Response

**Published:** 2023-10-23

**Authors:** Yunshun Chen

**Affiliations:** Walter and Eliza Hall Institute of Medical Research, Australia

We warmly thank the Reviewers for their positive assessment of our work, and for the numerous suggestions that helped us considerably improve the manuscript.

***When locally rerunning the code, outcomes are not fully reproducible. Most likely this is due to the fact the authors do not set random state for the code. The authors should consider fixing this.***

 Random seeds were set for those steps that involve randomness. In particular, we use set.seed(42) before running scDblFinder::scDblFinder() and monocle3::cluster_cells(). All the Suerat functions (e.g., RunPCA, RunUMAP) also use a fixed random seed by default. The outcomes are fully reproducible provided the same versions of all the packages are used. The results may slightly differ (e.g., the selection of starting node of the time-course trajectory) if different versions of one or more packages are used.

***Some lines of code present in the manuscript are missing on the Github repository. Is there any rational for this?***

 We thank the reviews for point this out. We have now added all the code required to run the workflow from start to finish.

***In the introduction, python-specific pipelines (e.g. scanpy) should also be referred to.***

 The scanpy reference has now been added to the introduction.

***When introducing the pseudo-bulk method to identify marker genes (3rd paragraph in the introduction), the authors mention that it has superior computational efficiency. Could the authors specify what they mean?***

 The advantages of the pseudo-bulk methods over single-cell level methods, including the computational efficiency, have been explored and described in greater details in the cited publication [1].

***In the last paragraph of the introduction, the authors say that they present a “new” single cell workflow. In what sense this pipeline is novel?***

 All the existing software tools (e.g., monocle3, slingshot, etc.) perform trajectory analysis at the single-cell level. Meanwhile, most single-cell pseudo-bulk analyses are performed for group-wise comparisons (between samples, clusters, or experimental conditions). The single-cell workflow we present is novel in the way that it utilizes the advanced edgeR GLM framework in modelling pseudo-time effect and combines the single-cell level trajectory analysis with the pseudo-bulking strategy.

***In “reading the data” section, two for loops of the first gray box run from 1:5. However, to make it as general as possible for future users of the pipeline, 5 should be replace by length(samples) or length(targets).***

 We thank the reviewers for the kind suggestion. Since the number of samples is fixed at 5 throughout the manuscript, we think using ‘5’ explicitly would simplify the code a bit. However, we could certainly consider generalize the coding format in the future.

***In “reading the data” section, the step in which a dge_merged object is created is unnecessary. All the QC analysis presented later is done at the level of individual samples: working with the elements in the list dge_all would simplify several lines of code.***

 Since one of the samples (Puberty) was processed using a different version of mouse genome, subsetting by row is required for all five DGEList objects. And this is easier to deal with using the merged DGEList object.

***In “reading the data” section, in the last gray box the authors show number of genes. Maybe they could consider printing out also the number of reads per sample, since this is a common QC.***

 The number of reads per sample reflects the sequencing depth of that entire sample, which is of less interest for QC at single-cell level. The cell-level QC statistics, which we showed in Fig1, are often more informative.

***In the sub-section “Quality control”, the authors could elaborate a bit more on what each QC parameter is telling us. For example, high mitochondrial genes indicate damaged or dead cells.***

 We thank the reviewers for the comment. We have added some extra explanation on interpreting the QC metrics.

***In sub-section “Quality control”, the choice of thresholds (<500 number of genes, >10% mitochondria, thresholds for high number of genes) seem very arbitrary. The plots on Figure 1 should be modified to visualize better the effect of the different thresholds. In addition, a histogram of the log-number of reads per cell should be included as a QC. In addition, the y label in Figure 1 should be replaced: library size is not the same as number of counts per cell. In the caption, one should explicitly mention that each dot is a cell barcode.***

 These thresholds were chosen in the same way as in the original paper [2]. We did not visualize the thresholds in Fig1 since some of the thresholds were determined afterwards by examining the scatter plots in Fig1. We did not produce a histogram of the log-number of reads per cell as it does not provide extra information in addition to Fig1 for QC. The y-label is now renamed to ‘Number of reads’, and it is now explicitly mentioned in the caption that each dot is a cell.

***When each dataset is converted into a Seurat object, the parameters min.cells = 3 and min.features = 200 should be discussed. How relevant is the min.features parameter given the previous 500 threshold?***

 When each individual data is read into a Seurat object, the “min.cells = 3” and “min.features = 200” are the initial QC parameters adopted in a Seurat online vignette [3]. The “min.features” would not be relevant to the downstream analysis since a threshold of 500 is applied right after that. We have now revised that part of the manuscript to clarify that.

***In the “Standard Seurat analysis of individual sample” sub-sections, the authors should replace “the data of each sample” by the “raw counts of each sample” when describing the normalization step.***

 We thank the reviewers for the comment. We have revised the sentence accordingly.

***In the “Standard Seurat analysis of individual sample” sub-section, 30 PCs are used by default to perform the umap embedding. The authors should discuss why 30, and whether/why this is a good choice for all the libraries.***

 Here we use the first 30 PCs to be consistent with the analysis in Pal *et al*. 2021 [2]. In general, the downstream results are very robust on the number of PCs chosen provided it is large enough (>10). We have now added some extra comments to the manuscript.

***In addition, why do they use different resolution for each dataset for the cell clustering? What criteria is followed to decide on this? Maybe some Silhouette plot or Gap Statistics should be included?***

 As described in the manuscript, cell clustering resolution is carefully chosen for each sample so that distinct cell types are grouped into separate clusters. This process usually involves some trial and error with different resolution parameters so that the final clustering result agree with its UMAP visualization. We did not use any sophisticated methods or statistics to set resolution as this would make the workflow more complicated than it should be.

***For the sake of readability, the authors could consider including a sub-section header after the first gray box of the “removing potential doublets and non-epithelial cells” sub-section.***

 We thank the reviewer for the suggestion. However, the content after the first grey box is still part of the sub-section “removing potential doublets and non-epithelial cells”.

***The choice of clusters expressing Epcam seems arbitrary: why is cluster 0 included in pre-puberty***

 The mammary gland epithelium consists of three major subtypes: basal, luminal progenitor (LP) and mature luminal (ML). Using some other marker genes, we can confirm that the cluster 0 in pre-puberty is the basal population, which is a subpopulation within epithelium.

***why are not all the clusters included in puberty***

 In the puberty sample, cells in cluster 8 are stromal cells and they have low expression of Epcam. Cells in cluster 7 do express Epcam. However, these cells have much higher library size than cells in other clusters, and hence could be homotypic doublets. Therefore, cluster 7 is also removed.

***and why in adult cluster 4 is not included?***

 In the adult sample, cells in cluster 4 are stromal cells and they have low expression of Epcam.

***To select Epcam+ clusters a bit more quantitatively, a boxplot (or violin plot or heatmap) should be made displaying mean Epcam expression per cluster.***

 As mentioned above, even though Epcam is a typical signature gene of epithelium, we still need to examine some other markers to fully confirm the identities of different cell clusters. We intentionally do not include all the details as this would go way beyond the scope of this workflow. This part of the workflow is simply to demonstrate that users can subset their data and focus on the cell type of their interest. When users apply our workflow to their own single-cell data, the marker genes and subsetting strategies might be completely different to ours.

 The main focus of this workflow is to showcase a novel approach that combines the single-cell level pseudotime trajectory analysis with the pseudo bulking strategy followed by an edgeR-style time course analysis. This main part of the workflow would remain the same regardless of what cell type users are interested in and how these subsets are obtained.

***The Expcam expression range use to select epithelial cells should either be the same for the 5 samples or follow some trend as a function of time. Can the authors show this in a plot?***

 In general, the Epcam gene is highly expressed in the epithelial cell population (basal, luminal progenitor, and mature luminal cells) compared to other cell population. Here we identify epithelial cell clusters by examining the expression level of Epcam within each individual sample. However, the Epcam expression levels are not directly comparable between different samples.

***Did the authors try to re-cluster data after doublet removal? This might be impactful in some datasets (such as the Puberty one).***

 We did try re-clustering data after doublet removal. We did not show it in the manuscript as i) the clustering results are very similar as before, and ii) all the samples are integrated right after doublet removal so there is no use of the re-clustering results from individual sample.

***In “integrating epithelial cells of five samples”, why do we need to integrate the data using the anchor-based method? How does the UMAP of all combined epithelial cells look like without the anchor-based strategy?***

 If no integration is performed, a clear batch (sample) effect can be observed on UMAP (i.e., cells of the same cell population are separated by sample).

***The author listed some batch-correction methods in the introduction, but here they do not explain the choice of this anchor-based method. Could they comment on this?***

 We use the anchor-based method as we adopt the Seurat workflow for all the single-cell analyses in the manuscript and Seurat uses the anchor-based integration method by default. We now added some comments on other integration methods as well.

***Why do the authors use the min.cells = 3 parameter in the first gray box of the “integrating epithelial cells of five samples”?***

 The ‘min.cells = 3’ is used to remove lowly expressed genes as these genes are not of any biological interest here. The same threshold is also used in the Seurat online vignette [4] although it is somewhat *ad hoc* and can be adjusted depending on the data. We added some extra comments on that to the manuscript.

***Which criteria does the “findIntegrationAnchors” to select features?***

 The details of the feature selection criteria in “findIntegrationAnchors” is explained in the Seurat paper [5]. We did not include it as it is beyond the scope of the manuscript.

***As before, 30 PC are used. Additionally, clustering is done using a resolution of 0.2. How did the authors decide of these values?***

 As mentioned above, we use the first 30 PCs to be consistent with the analysis in Pal *et al*. 2021 [2]. The choice of the number of PCs (i.e., 30) is also consistent with the Seurat online vignette [4]. As for the cell clustering resolution, we choose 0.2 after experimenting with different resolution parameters. This is because under this resolution the three major epithelial subpopulations, two intermediate cell clusters, and a small group of stroma cells can be clearly separated in distinct cell clusters. We added some extra comments to the manuscript.

***While rerunning the code to generate Fig. 3, a slightly different version of the UMAP was obtained. This leads to define different cell clusters and generate list of markers gene for downstream. Maybe it would be good to set a random seed in the umap or FindNeighbors to make the results from this pipeline robust.***

 We thank the reviewers for the kind suggestions. As mentioned before, we set random seeds for analysis steps that involve randomness. In addition, all the Seurat functions such as RunPCA and RunUMAP use a fixed random seed by default. The outcomes are fully reproducible if the same versions of R and all the R packages, as well as the same operating system, are used. The whole analysis workflow itself is very robust, and the results (e.g., marker genes, UMAP visualization, etc.) would not be significantly different if different versions of R or R packages are used.

***In “cell type identification”, the authors should consider extending the gene marker list. Why were specifically these markers selected? Csn3 and Elf5 are markers for luminal alveolar cells that are particularly active during lactation and not in the progenitor stage. Also Prlr is considered to be involved in milk production. What about performing Gene Ontology on the clusters?***

 The mouse mammary gland epithelium has been well studied in the literature. We have extensive lists of marker genes for each of the three major epithelial subpopulations (basal, LP and ML) from previous bulk RNA-seq experiments [6]. In fact, these hand-picked marker genes are all typical markers of the corresponding epithelial major cell populations, and they all appear in the lists of DE genes of the published study. There is no need to perform GO analysis as we can already identify the three major epithelial subpopulations with great confidence.

***Maybe cluster 5 should be removed from downstream analysis since it is stromal cells contamination?***

 We thank the reviewers for the suggestion. The cluster 5 is stroma contamination, but it only contains a total of 18 cells compared to ~22,000 cells from all five samples. The effect of having this small cell cluster in the downstream analysis is negligible. Therefore, we did not remove it for simplicity.

***What is the definition of a “key point” along a trajectory?***

 We thank the reviewers for pointing this out. A more precise word for this should be “principal nodes” as used by the monocle3 authors. The principal nodes (or points) include roots, leaves and branch points in the graph (the trajectory is a graph), they are identified by learn_graph() function in monocle3.

***How does pseudotime estimation compare to the embryo stage of origin? A scatter plot would be very informative.***

 The information of the pseudo-time estimation under each embryo stage of origin is summarized in the MDS plots (Fig 7).

***In section “constructing pseudo-bulk profiles”, the authors aggregate cells with the same combination of sample and cluster. However, this goes against the anchor-based integration method. Could the authors discuss why they do not use pseudotime intervals to aggregate cells with similar pseudotime values?***

 Aggregating cells with the same combination of sample and cluster is the traditional way of constructing pseudo-bulk profiles [1], regardless of the integration method. We don’t see why this is considered “against the anchor-based integration method”. Using pseudotime intervals could be an alternative way for constructing pseudo-bulk samples. We do not use it here because the current approach (sample-cluster combination) is more straightforward, and it provides enough information for assessing the biological variation between different samples.

***In the “design matrix” sub-section, could the authors discuss the structure of the design parameter? What are Z1, Z2 and Z3?***

 Response: Z1, Z2, and Z3 are the three columns of the matrix that represents the family of piecewise-cubic splines generated by splines::ns(). Their values do not have any particular meaning in general. More detailed description has been added to the manuscript regarding the interpretation of the three spline coefficients.

***What does CPM stand for in Figures 8 and 9?***

 CPM stands for ‘count-per-million’. The figure captions are now revised to explain what CPM means.

***It would be beneficial to explain better what is the y-axis of Figure 9. This section is in general very challenging to follow for anyone that does not have experience in bulk differential expression analysis. At least, a proper definition of “dispersion” should be given.***

 As stated in caption of Fig 9 (now Fig 10), the y-axis represents the quarter-root of the QL dispersion. We now added an extra sentence describing what QL dispersion is. References were also given for readers who want to know more about the details. Note that reads are expected to have some prior knowledge in both single-cell RNA-seq and bulk RNA-seq analysis to effectively follow the entire workflow.

***In Figure 10, it would be useful to color the black dots according to sample of origin to better understand the trends.***

 We thank the reviewers for this comment. However, this particular analysis and its results focus on pseudotime rather than sample of origin. Adding irrelevant information such as sample of origin to Fig 10 (now Fig11) would make the plots less straightforward.

***Some of the genes shown in Figure 10 should also be displayed in the UMAP obtained with integration of epithelial cells to appreciate better gene expression patterns along pseudo-time and along cell types.***

 We thank the reviewers for this suggestion. The plots in Fig 10 (now Fig 11) have already illustrated the strong association between gene expression and pseudo-time.  We don’t see the necessity of displaying them again in the UMAP. In addition, this workflow was structured in the way that every step after “Pseudo-bulk time course analysis with edgeR” is performed in edgeR at the pseudo-bulk level.

***In the Gene ontology and KEGG analysis subsections, it is not clear whether the GO is performed on the genes that exhibit a significant linear increase or decrease with pseudotime. Could the authors clarify this?***

 We did mention in the manuscript that: “To perform a GO analysis, we apply the goana function to the above test results”. To avoid confusion, we also used a different object ‘res_2’ for storing the testing results.

***In the discussion, the authors mention that their analysis revealed new insights specific to early developmental stages of the mammary gland. Could the authors elaborate on those?***

 The new insights refer to the discoveries of genes and pathways that exhibit continuous changes along the epithelial lineage, which is now specifically mentioned in the discussion now.

***We would advise the authors to load all the required libraries at the beginning of the pipeline.***

 We thank the reviewers for this suggestion. We decided to load each package when it is first used. This would give users a better understanding of which part of the workflow is conducted using which specific R package(s). We also added a section of “Package installation” at the start to tell users what packages are required for running through the workflow. Once all the required packages are installed, there would be no trouble loading them later on.

**References:**

 1. H. L. Crowell, *et al*. muscat detects subpopulation-specific state transitions from multi-sample multi-condition single-cell transcriptomics data. *Nat. Commun*., 11 (1):6077, 2020.

 2. B. Pal, *et al*. Single cell transcriptome atlas of mouse mammary epithelial cells across

 development. Breast Cancer Res., 23(1):69, 2021.

 3. https://satijalab.org/seurat/articles/pbmc3k_tutorial.html

 4. https://satijalab.org/seurat/articles/integration_introduction

 5. Y. Hao, *et al*. Integrated analysis of multimodal single-cell data. *Cell*, 184(13):3573–3587 e29, 2021.

 6. J. M. Sheridan, *et al*. A pooled shRNA screen for regulators of primary mammary stem and progenitor cells identifies roles for Asap1 and Prox1. *BMC Cancer* 15 (1): 221. 2015

# Reviewer report: Reviewer response for version 1

**DOI:** 10.5256/f1000research.147104.r190774

**Published:** 2023-08-25

**Authors:** Koen Van den berge

**Affiliations:** 1 Statistics and Decision Sciences, Janssen R&D, Beerse, Belgium

This article develops a method to discover genes whose gene expression is associated with a dynamic process, represented as a trajectory; a timely and critical contribution that is useful for the community. While several methods exist for this, the authors develop a procedure that is able to deal with multi-sample single-cell RNA-sequencing data. This extension is important, however some steps in the workflow may be improved upon.

**Major comments:**

- The workflow R script on GitHub does not contain several chunks of code of the workflow. It seems like many of the chunks that require a larger amount of time are not included, and need to be copied from the paper to the R script if one would like to reproduce the analysis. Please ensure the script is complete to increase ease of reproducibility.
- The authors use a 'standard' Seurat workflow to process the dataset before showcasing their methodology. While I understand that the general workflow is considered standard and expanding on it too much would deviate from the focus of the article, the description of some of the steps is inaccurate or incomplete. Across the manuscript, it would be good to add a bit more context. For example, for the first steps of the workflow, the 'NormalizeData' function does not simply log-normalize but divides the count by the total count for that cell, multiplies by a scale factor and then log-normalizes. Also, please mention the number of PCs being calculated, the clustering method being used and why sample-specific clustering resolutions being chosen for different samples.
- When following the workflow, I was surprised to see the authors pick a starting point for the trajectory in a region containing mainly cells from the later stages of development (pre-puberty/puberty/adult). The trajectory constructed in this way therefore goes from late stage (cluster 1) to early stage (cluster 4), back to late stage (cluster 2), back to early stage (cluster 3) and finally back to late stage cells (cluster 0). I therefore am suspicious of the biological relevance of this trajectory. Would a branching trajectory starting in the early stage not make more sense? Would the authors' method be able to handle such a setting? If not, an alternative dataset may be more useful, and this limitation should be clearly stated.
- I like the authors' push towards thinking about dealing with replication in trajectory-based differential expression analysis, but some steps seem rather crude:
  (a) the pseudo-bulking happens in the traditional way: for each combination of sample and cluster/cell type. These clusters are obtained in an unsupervised way, with no knowledge of the underlying trajectory and may therefore contain cells with very different pseudotimes. For example, cluster 3 has a group of cells at the left hand side of the UMAP (around -5 of first UMAP dimension), a region with a relatively low pseudotime, but most cells reside in a region with a high pseudotime (around +4 of first UMAP dimension). Would a trajectory-informed grouping of cells make more sense, e.g., making groups of cells based on binning pseudotime?
  (b) The pseudotime corresponding to each pseudobulk sample is obtained by averaging all cell-level pseudotimes. This seems simplistic, and I wonder if alternatives would be useful. First, the average may not be the best summary metric, given the distribution of pseudotimes within each of the groups (see figure I posted here https://github.com/jinming-cheng/TimeCoursePaperWorkflow/issues/1), and a median may be more appropriate. Alternatively, if possible, one idea would be to project the averaged/pseudobulked expression profile to the UMAP, and calculate the pseudotime of the projected point. In essence, instead of taking the average of pseudotime, one would project the average expression profile onto the trajectory.
- The authors construct a natural cubic spline and use its basis functions as covariates in edgeR. This is an efficient way of estimating smooth functions of pseudotime. Note, however, that the smoothness is fixed and is assumed to be identical for all genes. In smoothing, the smoothness is often controlled using a penalty parameter that is estimated using techniques like cross-validation. This does not happen here. The authors should acknowledge this limitation.

**Minor comments:**

- In general, the introduction seems very brief and it would be useful to add more context. A few examples:
  - Second paragraph of introduction: it would be helpful for unfamiliar readers to expand what is meant here with 'replicate samples'. The authors are likely thinking about different samples as obtained from different subjects. The pseudobulking approach they use may not be the best approach if the replicate samples may have been derived from the same subject. In addition, integration across samples may not always be necessary and whether or not to perform this should be carefully evaluated. Minimal sample effects may occur in e.g. studies using multiplexing. It would be good to add this nuance.
  - Third paragraph of introduction: It would be relevant to specify what is meant with 'the pseudo-bulk method'.
  - Fourth paragraph: When would trajectory inference be preferred? Expand how pseudotime is derived from a trajectory and why this is useful.
- The last paragraph of the introduction should be partly rephrased:
  - "The single-cell level analysis is performed in Seurat, and the trajectory analysis is conducted using monocle3":  both of these analyses are 'single-cell level analysis'.
  - "The analysis pipeline presented in this article can be applied to any scRNA-seq study with replicate samples." while this is true in theory, many datasets are not suitable for trajectory inference, as the biological context of a dataset may not constitute a 'dynamic system'.
- The paper mentions "The calculation of pseudotime, which indicates the distance between a cell and the starting cell in a trajectory, is conducted during the trajectory learning process", but this is inaccurate. The pseudotime is the distance between a cells' projection on the trajectory and the starting point of the trajectory, as measured along that trajectory. Current phrasing could be misunderstood as Euclidean distance between two points in e.g. the UMAP space.
- It may be useful to visualize the spline basis functions to allow the reader to gain intuition on what is happening.

Is the rationale for developing the new method (or application) clearly explained?

Yes

Is the description of the method technically sound?

Partly

Are the conclusions about the method and its performance adequately supported by the findings presented in the article?

Yes

If any results are presented, are all the source data underlying the results available to ensure full reproducibility?

Yes

Are sufficient details provided to allow replication of the method development and its use by others?

Yes

Reviewer Expertise:

Statistical omics

I confirm that I have read this submission and believe that I have an appropriate level of expertise to confirm that it is of an acceptable scientific standard, however I have significant reservations, as outlined above.

# Response

**Published:** 2023-10-23

**Authors:** Yunshun Chen

**Affiliations:** Walter and Eliza Hall Institute of Medical Research, Australia

We warmly thank the Reviewer for his positive assessment of our manuscript and fruitful comments that helped us to improve the study in the revised version.

***Major comments:***

***The workflow R script on GitHub does not contain several chunks of code of the workflow. It seems like many of the chunks that require a larger amount of time are not included, and need to be copied from the paper to the R script if one would like to reproduce the analysis. Please ensure the script is complete to increase ease of reproducibility.***

 We thank the reviewer for pointing this out. We agree that all the code required for the workflow shall be included in the R script on GitHub. We have now updated the R script on GitHub accordingly (https://github.com/jinming-cheng/TimeCoursePaperWorkflow). It now includes all chunks of R code that allows users to reproduce the analysis from start to finish.

***The authors use a 'standard' Seurat workflow to process the dataset before showcasing their methodology. While I understand that the general workflow is considered standard and expanding on it too much would deviate from the focus of the article, the description of some of the steps is inaccurate or incomplete. Across the manuscript, it would be good to add a bit more context. For example, for the first steps of the workflow, the 'NormalizeData' function does not simply log-normalize but divides the count by the total count for that cell, multiplies by a scale factor and then log-normalizes. Also, please mention the number of PCs being calculated, the clustering method being used and why sample-specific clustering resolutions being chosen for different samples.***

 We thank the reviewer for this comment. We have now revised the ‘standard’ Seurat workflow part of the manuscript and added more detailed descriptions to some of the steps. The number of PCs and the clustering method are now mentioned in the manuscript. We also added some explanation on how cell clustering resolutions were chosen for different samples.

***When following the workflow, I was surprised to see the authors pick a starting point for the trajectory in a region containing mainly cells from the later stages of development (pre-puberty/puberty/adult). The trajectory constructed in this way therefore goes from late stage (cluster 1) to early stage (cluster 4), back to late stage (cluster 2), back to early stage (cluster 3) and finally back to late stage cells (cluster 0). I therefore am suspicious of the biological relevance of this trajectory. Would a branching trajectory starting in the early stage not make more sense? Would the authors' method be able to handle such a setting? If not, an alternative dataset may be more useful, and this limitation should be clearly stated.***

 We thank the reviewer for this comment. Our trajectory analysis workflow is based on pseudotime rather than real time. The selection of the starting point depends on the biology or questions of interest, and it doesn’t need to agree with real time. As mentioned in the manuscript, we choose a starting point for the trajectory in the basal cluster since mammary stem cells are known to be enriched in the basal population and give rise to LP and ML cells in the epithelial lineage. We wish to study how gene expression profiles change along this epithelial lineage by using the concept of trajectory and pseudotime and then performing a time-course analysis. The workflow we present is very flexible in the way that users can choose their own starting point depending on their research question. Of course, one can subset the data, focus on one particular branch, and pick a starting point at an early stage.

***I like the authors' push towards thinking about dealing with replication in trajectory-based differential expression analysis, but some steps seem rather crude:***

- ***the pseudo-bulking happens in the traditional way: for each combination of sample and cluster/cell type. These clusters are obtained in an unsupervised way, with no knowledge of the underlying trajectory and may therefore contain cells with very different pseudotimes. For example, cluster 3 has a group of cells at the left hand side of the UMAP (around -5 of first UMAP dimension), a region with a relatively low pseudotime, but most cells reside in a region with a high pseudotime (around +4 of first UMAP dimension). Would a trajectory-informed grouping of cells make more sense, e.g., making groups of cells based on binning pseudotime?***

We thank the reviewer for the thoughtful comment. Yes, pseudo-bulking cells based on binning pseudotime could be an alternative method. For this workflow, we adopted the traditional pseudo-bulking approach because i) it is more straightforward, and ii) it allows us to assess the biological variation between different samples. If cells are grouped based on, say binning pseudotime, then each formed pseudo-bulk sample would contain cells from different biological samples of origin, making it harder to account for the variation between those samples in the analysis.

- ***The pseudotime corresponding to each pseudobulk sample is obtained by averaging all cell-level pseudotimes. This seems simplistic, and I wonder if alternatives would be useful. First, the average may not be the best summary metric, given the distribution of pseudotimes within each of the groups (see figure I posted here https://github.com/jinming-cheng/TimeCoursePaperWorkflow/issues/1), and a median may be more appropriate. Alternatively, if possible, one idea would be to project the averaged/pseudobulked expression profile to the UMAP, and calculate the pseudotime of the projected point. In essence, instead of taking the average of pseudotime, one would project the average expression profile onto the trajectory.***

We thank the reviewer for sharing the ideas and thoughts on this. Yes, we use the average of cellwise pseudotime as the pseudotime of that pseudo bulk sample for simplicity. We also tried using the median instead of the mean, and the results are very similar. We now added a comment to the manuscript discussing different ways of defining the pseudotime for the pseudo bulked samples. Projecting the averaged/pseudobulked expression profile back to the UMAP/trajectory sounds like a very interesting idea. However, there isn’t an easy way to do so without reconstructing the UMAP and the trajectory with the projected points included. This would make the workflow way more complicated than necessary.

***The authors construct a natural cubic spline and use its basis functions as covariates in edgeR. This is an efficient way of estimating smooth functions of pseudotime. Note, however, that the smoothness is fixed and is assumed to be identical for all genes. In smoothing, the smoothness is often controlled using a penalty parameter that is estimated using techniques like cross-validation. This does not happen here. The authors should acknowledge this limitation.***

 We appreciate the reviewer's comment. Nevertheless, it's worth noting that the edgeR pipeline requires a single design matrix for all genes. Controlling the smoothness using a penalty parameter requires constructing gene-specific design matrices for different genes in the data, a task that is currently impractical.

***Minor comments:***

***In general, the introduction seems very brief and it would be useful to add more context. A few examples:***

***- Second paragraph of introduction: it would be helpful for unfamiliar readers to expand what is meant here with 'replicate samples'. The authors are likely thinking about different samples as obtained from different subjects. The pseudobulking approach they use may not be the best approach if the replicate samples may have been derived from the same subject. In addition, integration across samples may not always be necessary and whether or not to perform this should be carefully evaluated. Minimal sample effects may occur in e.g. studies using multiplexing. It would be good to add this nuance.***

 We thank the reviewer for the comment. Yes, the 'replicate samples' means different biological replicate samples. We have revised that sentence to avoid confusion. In this workflow, the five samples we used were from five different mice (i.e., not from the same subject).

***- Third paragraph of introduction: It would be relevant to specify what is meant with 'the pseudo-bulk method'.***

 A description of the ‘pseudo-bulk method’ have been added to the manuscript.

***- Fourth paragraph: When would trajectory inference be preferred? Expand how pseudotime is derived from a trajectory and why this is useful.***

 We mentioned that the trajectory inference is useful for studies that focus on cell differentiation or cell type development. The details of how pseudotime is derived from a trajectory and why it is useful are covered in the section “Trajectory analysis with monocle3” later on.

***The last paragraph of the introduction should be partly rephrased:***

***- "The single-cell level analysis is performed in Seurat, and the trajectory analysis is conducted using monocle3":  both of these analyses are 'single-cell level analysis'.***

***- "The analysis pipeline presented in this article can be applied to any scRNA-seq study with replicate samples." while this is true in theory, many datasets are not suitable for trajectory inference, as the biological context of a dataset may not constitute a 'dynamic system'.***

 We thank the reviewer for the above two comments. We have now revised the last paragraph of the introduction accordingly.

***The paper mentions "The calculation of pseudotime, which indicates the distance between a cell and the starting cell in a trajectory, is conducted during the trajectory learning process", but this is inaccurate. The pseudotime is the distance between a cells' projection on the trajectory and the starting point of the trajectory, as measured along that trajectory. Current phrasing could be misunderstood as Euclidean distance between two points in e.g. the UMAP space.***

 In the manuscript, we did specifically mention that this is “the distance between a cell and the starting cell in a trajectory”.

***It may be useful to visualize the spline basis functions to allow the reader to gain intuition on what is happening.***

 We thank the reviewer for this thoughtful comment. We have provided the visualization of fitted spline curves in the format of line graphs in Fig 11, which gives reader some intuition on what is happening. The visualization of the spline basis functions, on the other hand, would be less intuitive compared to this. This is mainly because the spline coefficients do not have any particular meaning.

# Response

**Published:** 2023-11-20

**Authors:** Gordon Smyth

**Affiliations:** Walter and Eliza Hall Institute of Medical Research, Australia

I would like to add to Yunshun Chen's comments regarding regression splines vs smoothing splines estimated by cross-validation (CV-splines). In our view, regression splines are more appropriate than CV-splines for this type of workflow. The first consideration is inferential purpose. Methods such as cross-validation or AIC are designed for prediction rather than for interpretation and tend to overfit data from an inferential of view by including terms that do not achieve statistical significance. Regression splines allow us to conduct rigorous and powerful likelihood ratio tests in edgeR, which would be impossible using penalized smoothing splines. A second consideration is practicality. CV-splines are best suited to larger datasets and using CV-splines for datasets with less than 20 residual df would not be reliable. The third consideration is generality. We chose a 3-dimensional basis for our regression splines. The preset dimension (or df) does limit the maximum complexity of curves that can be fitted. In essence, we are assuming that expression trends do not have multiple local maxima, something that would require 4 or more df to accommodate. This limitation was a deliberate decision because we think that more complex trends would rarely be of biological interest. Nevertheless, the workflow could easily accommodate regression splines with df=4 or df=5, which we think would be large enough to accommodate pretty much any smooth trend of biological interest. There is no limitation that the fitted curves for different genes must follow the same shape or have the same smoothness. Our 3-df regression splines can accommodate any trend shape from constant to monotonic to quadratic (up then down) to cubic (up, down, then up again). Classic smoothing splines define smoothness in terms of integrated squared second derivative and regression splines can take on any value for that measure.

# Reviewer report: Reviewer response for version 1

**DOI:** 10.5256/f1000research.147104.r190775

**Published:** 2023-08-03

**Authors:** Michael D Morgan

**Affiliations:** 1 Institute of Medical Sciences, School of Medicine, Medical Sciences and Nutrition, University of Aberdeen, Aberdeen, Scotland, UK

The workflow presented by Cheng *et al.,* seeks to provide a framework for differential gene expression analysis along an inferred cell trajectory from single-cell droplet RNA-sequencing data. Several code snippets are especially useful, for example the summarising and plotting expression of genes in a pathway, and downloading data directly from GEO.

 Numerous R packages are used in the workflow, and this presents the first barrier any potential user will face to running the workflow. To address this the authors should include clear instructions at the beginning of the workflow to install all of these different package dependencies, noting which are available through community resources, i.e. CRAN and Bioconductor, vs. those that require installation from work-in-progress repositories. Inclusion of sessionInfo(), while useful, isn't sufficient for newcomers to this type of analysis.

 The justification for the workflow is a little weak. This could be significantly strengthened by motivating the work by explaining why Monocle3 was selected over alternative packages that seek to reconstruct a pseudotermporal ordering, especially as the OSCA book describes pseutotime DGE analysis already. The motivation, and manuscript, could be further strengthened by providing concrete examples of the utility of the workflow, e.g. as a teaching tool, or introduction to DGE analysis along a pseudotime ordering for newcomers to the field.

 Several statements are made in the current manuscript that aren't strictly correct or are outdated: "an integration method is required to investigate all cells across all samples simultaneously" <- this ignores the common-place use of sample multiplexing to overcome batch effects. "As the cost of scRNA-seq continues to drop" <- what is the real world evidence that the cost of scRNA-seq is falling? Reagent costs rise with inflation (at best). If in reference to the cost of sequencing, this comes at the cost of requiring higher sequencing depths/throughput to achieve lower costs. This should be clarified.

 The authors use a dataset with 5 samples, each of which represents a different timepoint. They then proceed to batch integrate these time points, but do not highlight the confounding between development stage and batch which can lead to over-correction of batch effects and remove biological variation. Moreover, I would question whether this truly represents a replicated experiment when the cells from the same sample are not independent. This is important because these cells are pseudobulked by sample and cluster for the DGE analysis and hence are not independent replicates. The authors should discuss this and make it clear that these data are used for illustrative purposes only, and highlight these limitations.

 A comment on why the data were selected, and the specific samples would aid the clarity of the manuscript.

 The authors note that the samples used different feature annotation versions. The authors should note/describe what barriers this presents to downstream analyses, and if possible, provide a recommendation on how to resolve the issue. e.g. work from the sequence data and re-process using a harmonised genome build.

 The authors use a series of thresholds for quality control of single cells. In reality QC thresholds are highly dependent on the study data, e.g. quiescent or small cells may normally have low RNA expression levels and metabolically active cells may have a high mitochondrial content. The data-dependency of QC thresholds should be noted clearly here. Likewise, the choice to remove cells with large numbers of genes has the potential to remove genuine cells. No justification is chose for these thresholds, and given that doublet detection is performed later it is not clear what this achieves.

 The authors state a "standard Seurat analysis" was performed - it is not clear what this means, and is vague particularly for newcomers. This should be clarified with a concrete series of steps described.

 In the first Seurat-based analysis  the knn-graph and clustering steps are run on each sample separately. There needs to be some justification for these analyses steps otherwise the workflow is not a useful learning tool for newcomers to single-cell analysis.

 The authors subset the data using specific clusters. However, the selection of these clusters is not well justified. For instance, in the E18.5-epi sample selecting cluster 1 seems logical when cluster 3 is selected in sample P5. There needs to be a justification for why these clusters were selected otherwise it seems somewhat arbitrary and dependent on how hard one stares at the UMAPs.

 It should be noted that several steps in the workflow use algorithms with a random component. Consequently, I get different numbers of cells in each cluster. I suspect 2 possible sources: (1) differences in clustering perhaps due to a random element to the graph building, integration or clustering step, (2) random elements to the doublet detection. These should be stated clearly, and where a random element exists in the relevant algorithms, a seed is set to retain reproducibility between different runs of the same code and data. The downstream consequences are non-trivial. For instance, running the workflow on my local Mac, I detect 268 DEGs vs. the 3268 reported in the manuscript.

 Newcomers to the field and this workflow may be unfamiliar with which steps are computationally burdensome - these could be noted in the manuscript and accompanying code to alert users that patience is required at these steps, e.g. FindIntergrationAnchors().

 The authors construct a single linear trajectory through the selection EpCam+ cells. Does it even make sense to have a single linear trajectory for a differentiation process that includes a bifurcation point? This analysis doesn't seem appropriate for the composition of the data - separating the two lineages would make more sense especially for the subsequent DGE analysis.

 The steps to build the spline should be explained in more detail. e.g. what does the QR decomposition do, what is it's purpose/necessity in terms of finding the smooth linear trends over samples w.r.t. pseudotemporal ordering.

Is the rationale for developing the new method (or application) clearly explained?

Partly

Is the description of the method technically sound?

Partly

Are the conclusions about the method and its performance adequately supported by the findings presented in the article?

Partly

If any results are presented, are all the source data underlying the results available to ensure full reproducibility?

Yes

Are sufficient details provided to allow replication of the method development and its use by others?

Partly

Reviewer Expertise:

Computational biology, single-cell, genetics, immunology

I confirm that I have read this submission and believe that I have an appropriate level of expertise to confirm that it is of an acceptable scientific standard, however I have significant reservations, as outlined above.

# Response

**Published:** 2023-10-23

**Authors:** Yunshun Chen

**Affiliations:** Walter and Eliza Hall Institute of Medical Research, Australia

We warmly thank the Reviewer for his positive assessment of our work and for the constructive comments that helped us enhance the strength of the manuscript.

***Numerous R packages are used in the workflow, and this presents the first barrier any potential user will face to running the workflow. To address this the authors should include clear instructions at the beginning of the workflow to install all of these different package dependencies, noting which are available through community resources, i.e. CRAN and Bioconductor, vs. those that require installation from work-in-progress repositories. Inclusion of sessionInfo(), while useful, isn't sufficient for newcomers to this type of analysis.***

 We thank the reviewer for pointing this out. We totally agree with the reviewer that clear instructions of package installation should be given at the start of the workflow. We have now added a new section of “Package installation” at the start of the workflow.

***The justification for the workflow is a little weak. This could be significantly strengthened by motivating the work by explaining why Monocle3 was selected over alternative packages that seek to reconstruct a pseudotermporal ordering, especially as the OSCA book describes pseutotime DGE analysis already. The motivation, and manuscript, could be further strengthened by providing concrete examples of the utility of the workflow, e.g. as a teaching tool, or introduction to DGE analysis along a pseudotime ordering for newcomers to the field.***

 We thank the reviewer for the comment. We did try slingshot and noticed the results from slingshot are not as stable as those from monocle3. Note that we present this workflow not to popularize the use of a particular package, such as monocle3, for trajectory analysis. In fact, we mentioned in the manuscript that “ *alternative methods and packages can be used interchangeably with the ones implemented in this study, as long as they perform equivalent functions*.” The motivation of this workflow is to showcase a novel approach that utilizes the advanced edgeR GLM framework for a time-course analysis and combines the single-cell level trajectory analysis with the pseudo-bulking strategy.

***Several statements are made in the current manuscript that aren't strictly correct or are outdated: "an integration method is required to investigate all cells across all samples simultaneously" <- this ignores the common-place use of sample multiplexing to overcome batch effects.***

 The integration analysis not only overcomes batch effects, but also accounts for sample effects when assessing different biological samples simultaneously. The sample multiplexing strategy only adjusts the former but not the latter.

***"As the cost of scRNA-seq continues to drop" <- what is the real world evidence that the cost of scRNA-seq is falling? Reagent costs rise with inflation (at best). If in reference to the cost of sequencing, this comes at the cost of requiring higher sequencing depths/throughput to achieve lower costs. This should be clarified.***

 We thank the reviewer for the comment. The overall cost of kits has remained similar but the actual cost per sample has significantly decreased due to the advent of multiplexing technologies such as CellPlex and the Flex assay. We have now clarified that in the manuscript.

***The authors use a dataset with 5 samples, each of which represents a different timepoint. They then proceed to batch integrate these time points, but do not highlight the confounding between development stage and batch which can lead to over-correction of batch effects and remove biological variation. Moreover, I would question whether this truly represents a replicated experiment when the cells from the same sample are not independent. This is important because these cells are pseudobulked by sample and cluster for the DGE analysis and hence are not independent replicates. The authors should discuss this and make it clear that these data are used for illustrative purposes only, and highlight these limitations.***

 We appreciate the comment from the reviewer. Yes, the 5 samples are from different timepoints, and hence the pseudo-bulk samples are not independent replicates. The MDS plot of all the pseudo-bulk samples further confirms the existence of the sample effects. To address this, we revised our downstream DE analysis and now incorporate the sample effects into the design matrix. Accounting for the sample effects significantly increases the statistical power of the time-course analysis. As expected, we now detect more genes significantly associated with pseudotime.

***A comment on why the data were selected, and the specific samples would aid the clarity of the manuscript.***

 We select this data as we wanted to study the epithelial lineage by constructing a trajectory that models dynamic cellular changes along the lineage. It is also because this is a study we are very familiar with. In general, one could choose any single-cell experiments aimed at studying dynamic changes along a specific path, whether it involves cell differentiation or the development of cell types.

***The authors note that the samples used different feature annotation versions. The authors should note/describe what barriers this presents to downstream analyses, and if possible, provide a recommendation on how to resolve the issue. e.g. work from the sequence data and re-process using a harmonised genome build.***

 We thank the reviewer for the comment. In general, the same cellranger reference build is preferred for consistency, although the effect on the downstream analysis is negligible. We have added a note to the manuscript in this regard.

***The authors use a series of thresholds for quality control of single cells. In reality QC thresholds are highly dependent on the study data, e.g. quiescent or small cells may normally have low RNA expression levels and metabolically active cells may have a high mitochondrial content. The data-dependency of QC thresholds should be noted clearly here.***

 We agree with the reviewer that QC thresholds shall be considered carefully depending on the study data. We choose these thresholds in the workflow because our main focus is the epithelial cell population which contains decent amount of RNA and also with low mitochondrial content if healthy. We have added a note to the manuscript to clarify that the QC thresholds are data dependent.

***Likewise, the choice to remove cells with large numbers of genes has the potential to remove genuine cells. No justification is chose for these thresholds, and given that doublet detection is performed later it is not clear what this achieves.***

 Even though a separate doublet detection analysis is performed using scDblFinder, we notice from our own practise that the combination of both doublet detection and the removal of cells with large counts works the best. This approach is also adopted in Seurat single-cell analysis vignettes [1].

***The authors state a "standard Seurat analysis" was performed - it is not clear what this means, and is vague particularly for newcomers. This should be clarified with a concrete series of steps described.***

 A standard Seurat analysis refers to the standard way of analysing a scRNA-seq data using the Seurat package. We have now revised the ‘standard’ Seurat workflow part of the manuscript and added more detailed descriptions to some of the steps. We also refer the readers to Seurat online vignettes for more details.

***In the first Seurat-based analysis the knn-graph and clustering steps are run on each sample separately. There needs to be some justification for these analyses steps otherwise the workflow is not a useful learning tool for newcomers to single-cell analysis.***

 We performed standard Seurat analysis on each individual sample separately to get some general idea of each one of them. This is also needed for the downstream analysis such as subsetting the epithelial cell population. 

 Note that this workflow is not a learning tool for someone who is completely new to single-cell analysis. Even the OSCA book [2] put doublet detection and trajectory analysis in the “Advanced” section (and also the pseudobulk DE analysis in the “Multi-sample” section after “Advanced”).

***The authors subset the data using specific clusters. However, the selection of these clusters is not well justified. For instance, in the E18.5-epi sample selecting cluster 1 seems logical when cluster 3 is selected in sample P5. There needs to be a justification for why these clusters were selected otherwise it seems somewhat arbitrary and dependent on how hard one stares at the UMAPs.***

 As mentioned in the manuscript, we are mostly interested in the epithelial cell population which is typically marked by the Epcam gene. However, some other markers of basal, LP and ML cell populations may also be examined and considered. We have now revised that section accordingly.

***It should be noted that several steps in the workflow use algorithms with a random component. Consequently, I get different numbers of cells in each cluster. I suspect 2 possible sources: (1) differences in clustering perhaps due to a random element to the graph building, integration or clustering step, (2) random elements to the doublet detection. These should be stated clearly, and where a random element exists in the relevant algorithms, a seed is set to retain reproducibility between different runs of the same code and data. The downstream consequences are non-trivial. For instance, running the workflow on my local Mac, I detect 268 DEGs vs. the 3268 reported in the manuscript.***

 We appreciate the reviewer’s comment. We are fully aware of the random component in the workflow. Therefore, random seeds were set for the analysis steps that involve randomness. The outcomes are fully reproducible if the same versions of R and all the required R packages, as well as the same operating system, are used. The reason that the reviewer got different results are mostly due to the use of different versions of R or R packages. In addition, there is one step that may require a manual inspection if different versions of software are used, that is the selection of the starting node. This is because the node numbers may vary depending on the version of monocle3 used, and using the same node number as the starting node may lead to unexpected results. We have commented on this in the manuscript.

***Newcomers to the field and this workflow may be unfamiliar with which steps are computationally burdensome - these could be noted in the manuscript and accompanying code to alert users that patience is required at these steps, e.g. FindIntergrationAnchors().***

 We thank the reviewer for the comment. The running time depends on the computational environment and resources as well as the size of the data when running the workflow. We have now included comments to notify users about the expected time needed to complete these steps.

***The authors construct a single linear trajectory through the selection EpCam+ cells. Does it even make sense to have a single linear trajectory for a differentiation process that includes a bifurcation point? This analysis doesn't seem appropriate for the composition of the data - separating the two lineages would make more sense especially for the subsequent DGE analysis.***

 We constructed this single linear trajectory based on the mammary gland epithelial lineage, where mammary stem cells (enriched basal cell population) give rise to luminal progenitor and then become mature luminal. It is not a bifurcating process.

***The steps to build the spline should be explained in more detail. e.g. what does the QR decomposition do, what is it's purpose/necessity in terms of finding the smooth linear trends over samples w.r.t. pseudotemporal ordering.***

 We thank the reviewer for this comment. The QR decomposition was used to re-parametrize the design matrix so that the first coefficient Z1 represents the linear trend in pseudotime. This would allow us to identify genes of which the expression levels increase or decrease along pseudotime in general. Making the DE results ‘directional’ is essential for GO and KEGG pathway analysis performed later on. We have now added more detailed explanation accordingly.

**References:**

 1. https://satijalab.org/seurat/articles/pbmc3k_tutorial.html

 2. https://bioconductor.org/books/release/OSCA/
